# Phospho-Tyr705 of STAT3 is a therapeutic target for sepsis through regulating inflammation and coagulation

**DOI:** 10.1186/s12964-020-00603-z

**Published:** 2020-07-08

**Authors:** Shunyao Xu, Xiaojun Pan, Lingjie Mao, Hao Pan, Wenwei Xu, Yufeng Hu, Xueshu Yu, Zhiqiang Chen, Songzan Qian, Yincai Ye, Yueyue Huang, Jingye Pan

**Affiliations:** 1 Department of Intensive Care Unit, The First Affiliated Hospital of Wenzhou Medical University, Nanbaixiang road, Wenzhou, Zhejiang 325000 P.R. China; 2 Wenzhou Medical University, Wenzhou, Zhejiang P.R. China; 3 Department of Blood Transfusion, The First Affiliated Hospital of Wenzhou Medical University, Wenzhou, Zhejiang P.R. China

**Keywords:** Sepsis, STAT3, Inflammation, Coagulation

## Abstract

**Background:**

Sepsis is an infection-induced aggressive and life-threatening organ dysfunction with high morbidity and mortality worldwide. Infection-associated inflammation and coagulation promote the progression of adverse outcomes in sepsis. Here, we report that phospho-Tyr705 of STAT3 (pY-STAT3), not total STAT3, contributes to systemic inflammation and coagulopathy in sepsis.

**Methods:**

Cecal ligation and puncture (CLP)-induced septic mice were treated with BP-1-102, Napabucasin, or vehicle control respectively and then assessed for systemic inflammation, coagulation response, lung function and survival. Human pulmonary microvascular endothelial cells (HPMECs) and Raw264.7 cells were exposed to lipopolysaccharide (LPS) with pharmacological or genetic inhibition of pY-STAT3. Cells were assessed for inflammatory and coagulant factor expression, cell function and signaling.

**Results:**

Pharmacological inhibition of pY-STAT3 expression by BP-1-102 reduced the proinflammatory factors, suppressed coagulation activation, attenuated lung injury, alleviated vascular leakage and improved the survival rate in septic mice. Pharmacological or genetic inhibition of pY-STAT3 diminished LPS-induced cytokine production in macrophages and protected pulmonary endothelial cells via the IL-6/JAK2/STAT3, NF-κB and MAPK signaling pathways. Moreover, the increase in procoagulant indicators induced by sepsis such as tissue factor (TF), the thrombin-antithrombin complex (TAT) and D-Dimer were down-regulated by pY-STAT3 inhibition.

**Conclusions:**

Our results revealed a therapeutic role of pY-STAT3 in modulating the inflammatory response and defective coagulation during sepsis.

Video Abstract

## Background

Sepsis, a major burden on public health, is defined as a life-threatening organ dysfunction that results from a dysregulated host response to infection [1–3]. Despite advances in management in the past decades, sepsis is still the main cause of death in intensive care units with limited therapeutic opinions [4–7].

It is widely known that inflammation and coagulation are involved in the pathogenesis of sepsis in a mutually promoting manner [8–10]. Macrophages and endothelial cells play critical roles as responsive cells in aspects of inflammatory response and coagulative function during sepsis [11, 12]. A large number of studies have established that proinflammatory factors, such as IL-1β, IL-6 and TNF-α, function as mediators of the procoagulant process [13, 14]. The excessive cytokines from activated monocytes and endothelial cells immediately give rise to substantial release of TF that initiates the coagulation pathway, which in return leads to aggravated systemic inflammatory responses and tissue injury [15–17]. In addition, endothelial cells are in an unstable state with inflammatory mediators and innate immune elements and coagulation systems in sepsis [18]. The barrier function of endothelial cells is impaired which may result in adverse outcomes [19–21].

The IL-6/JAK2/STAT3 pathway is a major signaling pathway involved in modulating the inflammatory response during the pathogenesis of disease [22]. IL-6 is a pivotal cytokine with diverse physiological functions and increasing IL-6 level is one of the hallmarks of sepsis [23]. Moreover, IL-6 is reported to promote an increase in endothelial permeability in the inflammatory response [24]. Previous studies have shown that JAK2 and STAT3 are activated in an experimental mammalian sepsis model [25, 26]. The JAK2 inhibition can protect the animals from polymicrobial sepsis by modulating macrophage activation and cytokine production [27]. The suppression of STAT3 activity ameliorates lung inflammatory responses in LPS-induced acute lung injury (ALI) [28, 29]. However, mice with a conditional deletion of STAT3 in macrophages or endothelial cells are susceptible to LPS-induced septic shock associated with increased production of cytokines and adverse survival [30, 31]. To determine the intrinsic mechanism, we designed in vitro experiments utilizing macrophages and endothelial cells and in vivo experiments with cecal ligation and puncture (CLP)-induced septic mice. As a result, our findings provide evidence that inhibition of phospho-Tyr705 of STAT3 (pY-STAT3), not total STAT3, can markedly limit the hyperactivation of the inflammatory response, suppress coagulation activation, protect endothelial barrier function, alleviate lung injury and improve the survival in septic mice.

## Methods

### Reagents and antibodies

LPS was purchased from Sigma (*Escherichia coli* O111:B4, L2630). BP-1-102 (BP) was from MedChemExpress (HY-100493) and Napabucasin (Na) was purchased from Topscience (T3218). Starch broth was purchased from Sigma (P0727). The following antibodies for western blotting and immunofluorescence were used: anti-STAT3 (CST, #9139), anti-p-STAT3 (CST, #9145), anti-ERK1/2 (CST, #4695), anti-p-ERK1/2 (CST, #4370), anti-β-actin (CST, #3700), anti-TF (Abcam, #ab151748), anti-PAR1 (Santa Cruz, #sc5605), anti-MMP9 (Proteintech, #10375–2-AP), anti-Ki67 (Abcam, #ab16667), anti-F4/80 (Santa Cruz, #sc26642), anti-VE-cadherin (Abcam, #ab33168), anti-α-E-catenin (Santa Cruz, #sc9988), anti-c-Jun (CST, #9165), anti-p-JNK (CST, #9255), anti-P38 (CST, #8690), anti-p-P38 (CST, #9216), anti-AKT (CST, #4691), anti-p-AKT (CST, #4060), anti-P65 (CST, #9936), anti-p-P65 (CST, #9936), anti-JAK2 (CST, #3230), anti-p-JAK2 (CST, #3776), anti-TNF-α (CST, #3707), anti-p-IKKα/β (CST, #9936), anti-IKKβ (CST, #9936), anti-IκBα (CST, #9936), anti-p-IκBα (CST, #9936), goat anti-rabbit HRP-conjugated polyclonal antibody (Bio-Rad, #1706515), goat anti-mouse HRP-conjugated polyclonal antibody (Bio-Rad, #1706516), anti-rabbit FITC (Abcam, #ab6717), anti-mouse PE (Abcam, #ab130774), anti-rabbit Alexa Fluor (Abcam, #ab150078) and DAPI (CST, #4083). The following ELISA kits were used: TNF-α (Multi Sciences, #EK282), IL-1β (Abcam, #197742), IL-6 (Multi Sciences, #EK206), CXCL10 (Multi Sciences, #EK268), TF (Abcam, #ab214091), PAI1 (Westang, #F11404), TAT (Westang, #F11582), D-Dimer (Westang, #F10354) and VE-cadherin (Abcam, #210968).

### Animals

Toll-like receptor 4 (TLR4) mutant male C57BL/10ScNJ mice (TLR4^mut^) were purchased from the Jackson Laboratory. Male C57BL/6 mice (8–10 weeks old) were purchased from Shanghai SLAC Laboratory Animal Co (Shanghai, China). All mice were maintained in a pathogen-free facility under an automated 12 h dark-light cycle at an ambient temperature of 23 ± 3 °C and a relative humidity of 55 ± 10%. Food and water were available ad lib. We conducted all animal care and experimentation with approval from Wenzhou Medical University Institutional Animal Care and Use Committees.

### Cecal ligation and puncture (CLP)

The sepsis mouse model was induced by CLP as previously described [32]. The mice were anesthetized with 1% sodium pentobarbital (0.1 ml/10 g body weight; Solarbio, Beijing, China) before the operation. After an abdominal incision, the cecum was identified, ligated at the terminal part, and punctured twice with a 21-gauge needle (Kindly, Shanghai, China) to gently squeeze a droplet of its feces. Then the cecum was returned to the abdominal cavity and the abdomen was sutured in two layers. A sham group was established similarly without ligation or puncture. Subsequently, all the mice were subcutaneously injected with 1 ml prewarmed saline for fluid resuscitation. BP (5 mg/kg) and Na (10 mg/kg) dissolved in vehicle (2.5% DMSO, 2.5% Tween-80 and 95% PBS) were administered intraperitoneally to mice 2 h before CLP (optimal dosage of inhibitors was measured previously). The CLP group was just intraperitoneally treated with vehicle as a control. The animals were closely assessed every 6 h for the following 4 days and euthanized at the moribund stage. Plasma samples and lung tissues were collected 24 h after CLP.

### ELISA analyses

The supernatant from in vitro cultured cells or the plasma from experimental mice was quantified using ELISA kits following the manufacturer’s protocols.

### Cell culture and stimulation

The murine macrophage cell line Raw264.7 (used within 10 passages) was purchased from American Type Culture Collection (ATCC) and cultured in Dulbecco’s modified Eagle’s medium (Gibco, Life Technologies, Germany) supplemented with 10% fetal bovine serum (FBS) (Sigma-Aldrich, St. Louis, MO, USA) and 1% penicillin-streptomycin (Solarbio, Beijing, China) at 37 °C in a 5% CO2 humidified chamber. Human pulmonary microvascular endothelial cells (HPMECs) were purchased from ScienCell Research Laboratories (Carlsbad, CA, USA) and cultured in the specialized medium (ScienCell, USA). Raw264.7 cells and HPMECs were pretreated with BP-1-102 (5 μM, dissolved in DMSO) for 2 h and then stimulated with LPS (1 μg/ml) for 0.5 or 6 h.

Peritoneal macrophages (PMs) were isolated from 6- to 7-week-old C57BL/10ScNJ and C57BL/6 mice as previously described [32]. Briefly, mice were intraperitoneally injected with 2 ml of starch broth (Sigma, USA), and then the peritoneal cavity was washed with RPMI medium (Gibco, Life Technologies, Germany) three times to isolate peritoneal exudate cells. After 2 h of culture, the adhered cells were used as macrophages. PMs were stimulated with LPS (1 μg/ml) for 6 h for further experiments.

### Lentivirus transfection

To genetically suppress phospho-Tyr705 of STAT3 (pY-STAT3), the Y705 phosphorylation STAT3 mutant (Y705A) in which Y705 was replaced with alanine was generated from the GV492 vector (GeneChem, Shanghai, China) containing Ubi-MCS-3FLAG-CBh-gcGFP-IRES-puromycin. The Raw264.7 cells were transfected following the manufacturer’s instructions (GeneChem, Shanghai, China).

### Tube formation assay

HPMECs that reached 80% confluency were pretreated with BP (5 μM, dissolved in DMSO) or DMSO as a control. After 2 h, the cells (15 × 10^4^/well) were digested with trypsin (0.25% with EDTA, Gibco) and respectively seeded in 24-well plates covered with 120 μl polymerized Matrigel (BD Biosciences). Then LPS (1 μg/ml) was added for 2 h, and images were collected with a light microscope.

### HPMECs permeability assay

HPMECs (20 × 10^4^/well) were seeded in the upper chamber of 24-well Tanswell plate (0.4 μm pore; NEST Biotechnology, Jiangsu). The cells were cultured until confluence and then pretreated with BP for 2 h. LPS (1 μg/ml) was added to the apical medium for 6 h. The medium supernatant of each group was collected. The apical medium was then replaced with 200 μl of HRP solution (50 ng/ml). After incubation for 1 h, the medium of the lower chamber was collected and the penetrating HRP was assessed using a colorimetric assay with O-phenylenediamine for 10 min incubation at 37 °C. The OD_405_ was measured by a microplate reader (Molecular Devices, Hercules, CA, USA). To check the indirect effect of the cytokines on junctions, we transferred the collected medium supernatant to the corresponding HPMECs that recently reached confluency for 6 h and then assessed cell permeability as described above.

### Cell counting Kit-8 (CCK-8) assay

A CCK-8 kit (Dojindo, Japan) was used to analyze the cell viability. Raw264.7 cells or HPMECs were seeded at 3000 cells per well in a 96-well plate and given the corresponding treatment. After 24 h, 20 μl of CCK8 solution was added for 2 h incubation at 37 °C. OD_450_ values were measured.

### Immunoblotting

Immunoblotting analysis was carried out as we previously described [32]. The ECL Chemiluminescent Reagent (Advansta, USA) was used to visualize the proteins and the bands were quantitated by ImageJ (NIH, USA).

### RT-PCR

Total RNA was extracted from cultured cells and lung tissues using the TRIzol reagent (Invitrogen, CA). Moloney murine leukemia virus reverse transcriptase (Invitrogen, USA) was used for cDNA synthesis according to the manufacturer’s protocols. The LightCycler (Roche Diagnostics, Germany) and SYBR Green PCR Master Mix (Roche Diagnostics, Germany) were applied to detect mRNA expression with primer pair sequences (Table 1).

**Table 1:** Primer sequences for RT-PCR

Gene	Forward primer	Reverse primer
Mouse GAPDH	GCACAGTCAAGGCCGAGAAT	GCCTTCTCCATGGTGGTGAA
Mouse IL-1β	TGCCACCTTTTGACAGTGATG	TTCTTGTGACCCTGAGCGAC
Mouse IL-6	GCCTTCTTGGGACTGATGCT	TGTGACTCCAGCTTATCTCTTGG
Mouse IL-10	TAAGGCTGGCCACACTTGAG	GTTTTCAGGGATGAAGCGGC
Mouse TNF-α	ACCCTCACACTCACAAACCA	ACCCTGAGCCATAATCCCCT
Mouse CXCL1	GGATGCCACAGGATTCCATA	GTGCCATCAGAGCAGTCTGT
Mouse CXCL10	CCAAGTGCTGCCGCTCATTTTC	TCCCTATGGCCCTCATTCTCA
Mouse MCP1	TAAAAACCTGGATCGGAACCAAA	GCATTAGCTTCAGATTTACGGGT
Mouse COX2	TGCACTATGGTTACAAAAGCTGG	TCAGGAAGCTCCTTATTTCCCTT
Mouse MMP1	TGTTTGCAGAGCACTACTTGAA	CAGTCACCTCTAAGCCAAAGAAA
Mouse MMP8	TGGTGATTTCTTGCTAACCCC	TACACTCCAGACGTGAAAAG
Mouse MMP9	GGACCCGAAGCGGACATTG	CGTCGTCGAAATGGGCATCT
Mouse ICAM1	TTCTCATGCCGCACAGAACT	TCCTGGCCTCGGAGACATTA
Mouse TF	GCCACCATCTTTATCATCCTCC	AGCCTTTCCTCTATGCCAAGC
Mouse PAR1	CGCAGCGTTTTACGGGAAC	CTGGATCGGATACACCACCG
Mouse tPA	AGATGAGCCAACGCAGACAA	AACTTCGGACAGGCACTGAG
Mouse PAI1	GACGTTGTGGAACTGCCCTA	TCGCTATTGGGCCACCATTT
Human GAPDH	GGAGCGAGATCCCTCCAAAAT	GGCTGTTGTCATACTTCTCATGG
Human IL-1β	AACCTCTTCGAGGCACAAGG	GTCCTGGAAGGAGCACTTCAT
Human IL-6	ACCCCCAATAAATATAGGACTGGA	AGAAGGCAACTGGACCGAAG
Human IL-10	TACGGCGCTGTCATCGATTT	TAGAGTCGCCACCCTGATGT
Human TNF-α	GCTGCACTTTGGAGTGATCG	ATGAGGTACAGGCCCTCTGA
Human MCP1	GATCTCAGTGCAGAGGCTCG	TTTGCTTGTCCAGGTGGTCC
Human CXCL1	CTGGCGGATCCAAGCAAATG	GCCCCTTTGTTCTAAGCCAG
Human CXCL10	GTGGCATTCAAGGAGTACCTC	TGATGGCCTTCGATTCTGGATT
Human COX2	CTGGCGCTCAGCCATACAG	CGCACTTATACTGGTCAAATCCC
Human TF	GCCACCATCTTTATCATCCTCC	AGCCTTTCCTCTATGCCAAGC
Human TM	AGCGAGGGTAGGGAGGACTTGA	CAGCAGCACTAGGAGGTGAGGT
Human EPCR	TTGACGAAGTTTCTGCCGCTAC	CCTGATGCCTCACATGATGGTT
Human PAI1	ACGTGGTTTTCTCACCCTATGG	CATGCCCTTGTCATCAATCTTG

### Lung histology analyses

Lung tissues were fixed in 4% paraformaldehyde, dehydrated, embedded, and sectioned at a thickness of 4 μm for H&E staining. A scale of 0 to 3 (0 denoted no injury; 1, mild; 2, moderate; 3, severe) was used to evaluate lung injury.

### Lung wet/dry weight ratio

The lungs were weighed immediately after separation and then dried in an incubator at 80 °C for 48 h to obtain wet/dry weight ratios.

### Lung vascular permeability assessment

Evans blue dye (EBD) was injected into the tail vein 2 h before euthanasia. Then the lungs were perfused with PBS and incubated in 1 ml of formamide at 37 °C for 24 h. Following centrifugation (1000 g for 10 min), the EBD in the supernatants was measured spectrophotometrically at 620 nm.

### Immunofluorescence staining

The lung sections were dewaxed and rehydrated, and antigens were retrieved. The primary antibodies were applied at 4 °C overnight, followed by fluorescent secondary antibody and nuclei staining.

Macrophages or HPMECs were fixed, permeabilized, and blocked. Then the slides were incubated with primary antibodies overnight at 4 °C followed by fluorescent secondary antibody and nuclei staining. Images are representative of three independent experiments.

### Statistics

Statistical analysis was carried out with SPSS software 20.0. The Kaplan-Meier method was used to analyze the survival rate of different groups. Categorized variables were compared by one-way ANOVA or Student’s t test. In all tests, *P* < 0.05 was considered statistically significant.

## Results

### Pharmacological inhibition of pY-STAT3, not total STAT3, improves survival and blunts systemic inflammation and coagulopathy in septic mice

**Fig. 1:**
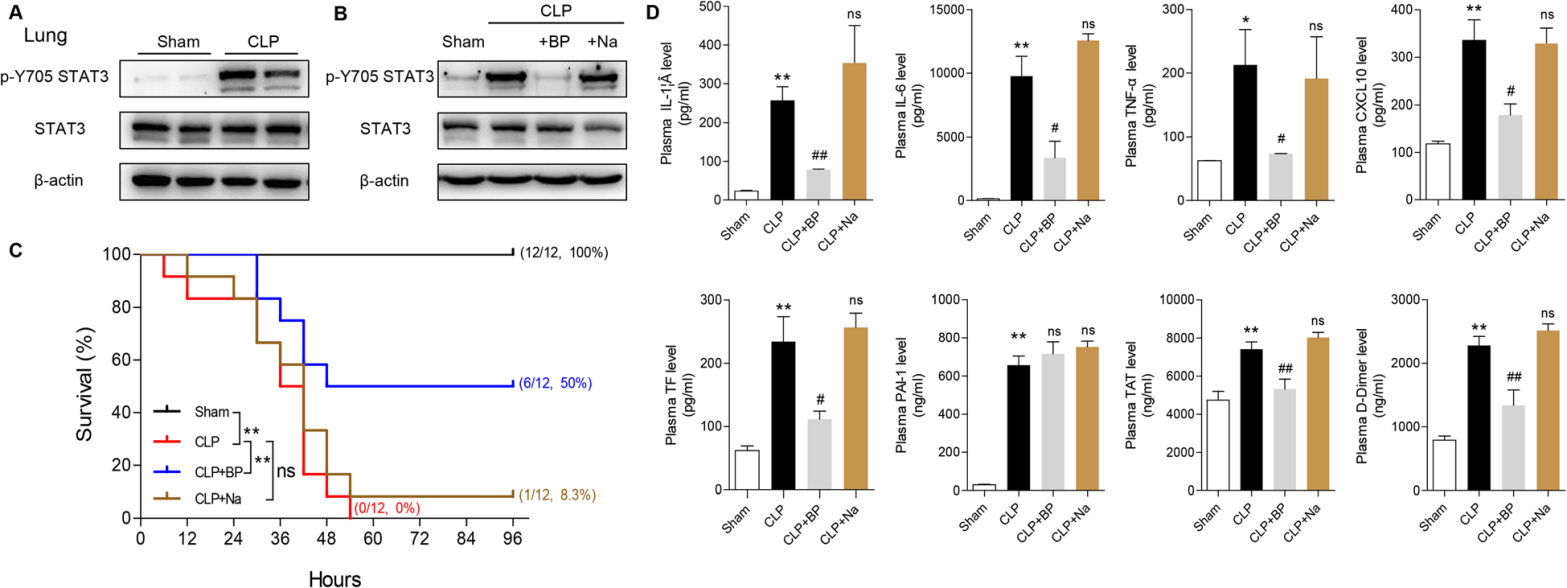
BP but not Na, protects mice from septic death, systemic inflammation and coagulopathy. **a** Immunoblotting analysis of pulmonary STAT3 and phospho-Y705-STAT3 in mice 24 h after CLP or shame operation. **b** Immunoblotting analysis of pulmonary STAT3 and pY-STAT3 in mice 24 h after CLP with BP or Na administration (*n* = 3 per group). **c** Administration of BP, but not Na prevented CLP-induced mouse death (*n* = 12 mice per group; Kaplan-Meier survival analysis). **d** IL-1β, IL-6, TNF-α, CXCL10, TF, PAI1, TAT and D-Dimer concentrations in plasma were measured 24 h after the CLP procedure by ELISA (*n* ≥ 5 per group; two-tailed Student’s test). **P < 0.01 and *P < 0.05 (CLP versus Sham); ##*P* < 0.01 and #*P* < 0.05 (CLP versus CLP + BP); ns indicates not significant

Given the importance of STAT3 in the inflammatory response, we found that the activation of pulmonary STAT3 in CLP-induced septic lung injury mice was significantly increased compared to that in sham group (Fig. 1a). As such, we chose BP-1-102 (BP, a specific STAT3-phospho-Tyr705 inhibitor) and Napabucasin (Na, a total STAT3 inhibitor) to assess the therapeutic potential of STAT3-targeting agents in septic mice. As a result, BP downregulated the elevated pY-STAT3 level in the mouse lung 24 h after CLP, while Na suppressed STAT3 expression and did not affect the pY-STAT3 level (Fig. 1b). In addition, treatment with BP, but not Na, protected mice against septic death (Fig. 1c), which was associated with reduced systemic proinflammatory and procoagulant-related factors (Fig. 1d). The elevated plasma levels of IL-1β, IL-6, TNF-α, CXCL10, TF, TAT and D-Dimer in CLP mice were downregulated by BP treatment. However, BP administration had no effect on the level of abnormal plasma plasminogen activator inhibitor 1 (PAI1) level.

### pY-STAT3 inhibitor attenuates CLP-induced lung injury in vivo

**Fig. 2:**
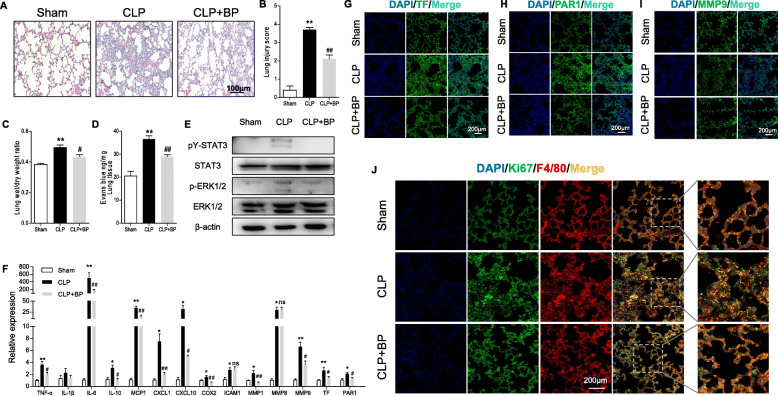
BP ameliorates lung injury in CLP-induced polymicrobial sepsis. Mice were pretreated with BP for 2 h and lung tissues were collected 24 h after cecal ligation and puncture procedures. **a** to **c** The lung tissue hematoxylin and eosin staining (**a**, scale bar: 100 μm), lung injury score (**b**), and wet/dry weight ratio (**c**) were analyzed (*n* ≥ 5 per group). **d** Evans blue dye extravasation of the lungs was analyzed spectrophotometrically (*n* = 4 per group). **e** Immunoblot analysis of STAT3 and ERK1/2 expression in lung tissues (*n* = 3 per group). **f** The mRNA levels of the indicated genes were assayed by RT-PCR (*n* ≥ 5 per group; results are the mean ± SEM; one-way ANOVA test). (**g** to **i**) TF (G, green), PAR1 (**h**, green), MMP9 (**i**, green) and nuclei (blue) immunofluorescent staining of lung sections imaged by laser confocal microscope (*n* ≥ 3 per group; scale bar: 200 μm). **j** Laser confocal microscope images of Ki67 (green), F4/80 (red) and nuclei (blue) staining in lung sections (*n* ≥ 3 per group; scale bar: 200 μm). **P < 0.01 and *P < 0.05 (CLP versus Sham); ##*P* < 0.01 and #*P* < 0.05 (CLP versus CLP + BP); ns indicates not significant

To study whether the pY-STAT3 inhibitor BP influences septic lung injury, we checked pulmonary function and some associated indicators in mice. Notably, the septic lung injury features, including an increase in inflammatory cell infiltrates, alveolar septal wall thickening, alveolar congestion and pulmonary edema, were attenuated by BP administration (Fig. 2a). Likewise, the elevated lung injury score (Fig. 2b) and wet/dry weight ratio (Fig. 2c) were reduced in BP-treated group compared with the CLP group. To evaluate pulmonary vascular barrier function, we observed endothelial permeability by an Evans blue dye assay. The results indicated that the CLP-induced lung endothelial hyperpermeability was blunted by BP treatment (Fig. 2d). Moreover, the immunoblots suggested that BP effectively inhibited CLP-induced pulmonary STAT3 and ERK1/2 phosphorylation (Fig. 2e). RT-PCR analysis indicated that TNF-α, IL-6, IL-10, MCP1, CXCL1, CXCL10, COX2, MMP1, MMP9, TF and PAR1 mRNA expression levels were increased in lung tissues during CLP compared with the sham group but decreased under BP administration except for IL-1β, ICAM1 and MMP8 mRNA levels (Fig. 2f). In addition, the images of TF, PAR1 and MMP9 fluorescent staining also partly verified the results of RT-PCR analysis (Fig. 2g, h and i). As macrophages play a vital part in the pathogenesis of sepsis, we performed F4/80 labeling for macrophages and Ki67 labeling for proliferating cells, which illustrated that the increase in macrophages infiltration in septic lung tissues was effectively decreased by BP treatment (Fig. 2j).

### pY-STAT3 inhibitor protects HPMECs from LPS damage

**Fig. 3:**
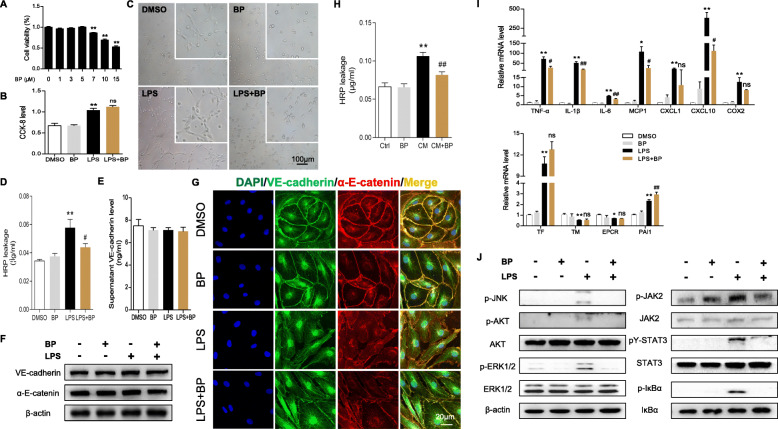
Effect of BP on endothelial permeability and inflammation in HPMECs. HPMECs were pretreated with BP for 2 h before LPS (1 μg/ml) challenge. **a**, **b** CCK-8 assay for measuring the HPMECs viability and proliferation (*n* = 6 per group; two-tailed Student’s test). **c** Screening for HPMEC tube formation in vitro after LPS stimulation for 2 h (*n* ≥ 3 per group; scale bar: 100 μm). **d** HPMECs permeability was measured 6 h after LPS challenge by observing HRP leakage through the Transwell system (0.4 μm pore). **e**, **f** ELISA and immunoblotting for VE-cadherin and α-E-catenin in HPMECs supernatant and lysates after LPS stimulation for 6 h (*n* = 3 per group). **g** VE-cadherin (green), α-E-catenin (red) and nuclei (blue) immunofluorescent staining of HPMECs visualized by laser confocal microscope (scale bar: 50 μm). **h** HPMECs permeability was measured 6 h after transferring medium supernatant by observing HRP leakage through the Transwell system (0.4 μm pore) (*n* = 6 per group). Conditioned medium (CM) indicates LPS-treated HPMECs medium, CM + BP indicates LPS + BP-treated HPMECs medium. **i** RT-PCR analysis of the indicated genes in HPMECs after LPS stimulation for 6 h (*n* = 4 per group; results are the mean ± SEM; one-way ANOVA test). **j** Immunoblotting of JAK2/STAT3, NF-κB and MAPK signaling molecules in HPMECs (n = 3 per group). **P < 0.01 and *P < 0.05 (LPS versus DMSO); ##*P* < 0.01 and #*P* < 0.05 (LPS versus LPS + BP); ns indicates not significant

The vascular system dysfunction characteristic of endothelial hyperpermeability is a key component in sepsis, so we investigated the effect of BP on endothelial cell function under LPS challenge. First, we tested the CCK-8 level of HPMECs with BP and determined that 5 μM was an ideal concentration (Fig. 3a). Then, we measured the HPMECs proliferation and found that LPS-induced cell proliferation was inhibited by BP (Fig. 3b). The tube formation assay indicated that the network formation of HPMECs was significantly enhanced under LPS stimulation but restrained by BP (Fig. 3c). To address whether pY-STAT3 inhibition has an effect on the regulation of vascular integrity with LPS, we evaluated HPMECs monolayer permeability and found that BP partly curbed the increase in HPMECs permeability induced by LPS (Fig. 3d). Since endothelial permeability is related to the control of cohesion and to the organization of intercellular junctions, we further examined the protein expression of junction proteins, including VE-cadherin and α-E-catenin, through ELISA and immunoblots. Interestingly, neither LPS nor BP affected on these two indicators in the cell supernatant and lysate (Fig. 3e and f). However, the integrity of VE-cadherin and α-E-catenin detected by immunofluorescence staining was disrupted and fractured between cells that were repaired by BP administration (Fig. 3g). This meant that LPS and BP did not induce changes in VE-cadherin and α-E-catenin protein expression but affected the localization of these proteins. To establish whether the hyperpermeability was indirectly due to the elevation in cytokines, we transferred LPS-treated HPMECs-conditioned medium (CM) and LPS + BP CM to newly cultured HPMECs. The results showed that LPS-treated CM led to more severe endothelial leakage than LPS + BP CM in HPMECs (Fig. 3h). Next, we measured the mRNA expression of proinflammatory mediators, including TNF-α, IL-1β, IL-6, IL-10, MCP1, CXCL1, CXCL10 and COX2, and coagulation-associated factors, including TF, thrombomodulin (TM), endothelial protein C receptor (EPCR) and PAI1. It showed that BP administration suppressed the LPS challenge-induced increase in the expression of most proinflammatory mediators except for that of CXCL1 and COX2, but had no effect on the four abnormal coagulation associated factors (Fig. 3i). Activation of inflammation signaling, such as the JAK2/STAT3, NF-κB, MAPK and AKT pathways, is necessary for TLR-mediated cytokine production. We evaluated JAK2/STAT3 pathway activation by measuring the phosphorylation of STAT3 and JAK2, NF-κB pathway activation by measuring the phosphorylation of IκBα, MAPK pathway activation by measuring the phosphorylation of ERK and JNK, and AKT pathway activation by measuring the phosphorylation of AKT and found that the phosphorylation of all these proteins was decreased by BP treatment in LPS-stimulated HPMECs (Fig. 3j). In summary, these findings collectively demonstrated that pY-STAT3 pharmacological inhibition prevented HPMECs from LPS damage through the JAK2/STAT3, NF-κB, MAPK and AKT pathways and enhanced endothelial integrity by cementing VE-cadherin and α-E-catenin localization and reducing cytokine levels.

### pY-STAT3 inhibitor reduces the surge in inflammatory mediators and TF in macrophages stimulated by LPS

**Fig. 4:**
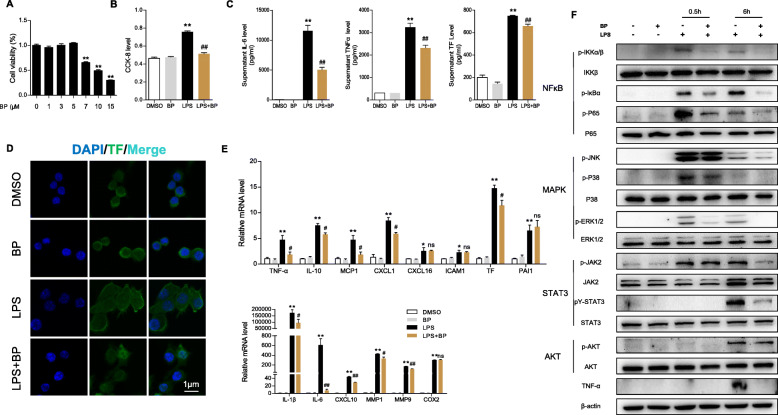
BP inhibits macrophage proinflammatory mediators and TF secretion by LPS stimulation. The Raw264.7 macrophage cell line was pretreated with BP for 2 h and then challenged with LPS (1 μg/ml) for 0.5 or 6 h. **a**, **b** CCK-8 assay was used to measure cell viability and proliferation (*n* = 6 per group; two-tailed Student’s test). **c** ELISA for IL-6, TNF-α, and TF in macrophage supernatant. **d** Laser confocal microscope screening of TF (green) and nuclei (blue) staining in macrophages (scale bar: 1 μm). **e** The mRNA expression of the indicated genes in macrophages was assayed by RT-PCR (*n* = 4 per group; results are the mean ± SEM; one-way ANOVA test). **f** The protein levels of JAK2/STAT3, NF-κB, MAPK and AKT signaling molecules in macrophages were assayed by immunoblotting (*n* = 3 per group). **P < 0.01 and *P < 0.05 (LPS versus DMSO); ##*P* < 0.01 and #*P* < 0.05 (LPS versus LPS + BP); ns indicates not significant

In light of the decrease in pulmonary macrophage infiltration, we aimed to explore the role of BP in activated macrophage regulation. Similarly, we tested the CCK-8 level of Raw264.7 cells with BP and chose 5 μM as the final concentration (Fig. 4a). As a result, LPS-induced macrophage proliferation was obviously inhibited by BP (Fig. 4b). The IL-6, TNF-α and TF levles in the cell supernatant were increased by LPS stimulation and diminished by BP treatment (Fig. 4c). The fluorescence intensity of TF in macrophages was enhanced by LPS induction but weakened in the presence of BP (Fig. 4d). We next examined the mRNA expression of TNF-α, IL-1β, IL-6, IL-10, MCP1, CXCL1, CXCL10, CXCL16, COX2, MMP1, MMP9, TF and PAI1, which suggested a similar trend to previous pulmonary results (Fig. 4e). Since the JAK2/STAT3, NF-κB, MAPK and AKT pathways are involved in activation of inflammation response and coagulation, we found that the LPS-challenged hyperactivation of JAK2/STAT3, NF-κB and MAPK pathways in macrophages was efficiently controlled by BP administration (Fig. 4f). Therefore, these results demonstrated that the pY-STAT3 inhibitor downregulated macrophage cytokine and TF production by the JAK2/STAT3, NF-κB, MAPK and AKT pathways.

### Genetic suppression of pY-STAT3 limits the LPS-induced hyperinflammatory response in macrophages

**Fig. 5:**
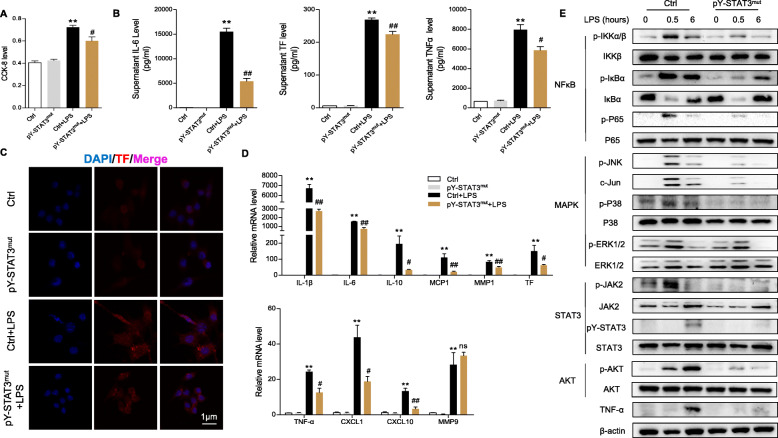
Genetic inhibition of pY-STAT3 abolishes LPS-challenged hyperinflammation in macrophages. The pY-STAT3 mutant Raw264.7 macrophage cell line was stimulated by LPS (1 μg/ml) for 0.5 or 6 h. **a** The proliferation of macrophages was analyzed by CCK-8 assay (*n* = 6 per group; two-tailed Student’s test). **b** ELISA for IL-6, TNF-α, and TF in macrophage supernatant. **c** Macrophages TF (red) and nuclei (blue) staining were imaged by laser confocal microscope (scale bar: 1 μm). **d** The levels of the indicated mRNAs in macrophages were assayed by RT-PCR (*n* = 4 per group; results are the mean ± SEM; one-way ANOVA test). **e** Immunoblotting analysis for JAK2/STAT3, NF-κB and MAPK signaling molecules in macrophages (*n* = 3 per group). **P < 0.01 and *P < 0.05 (control + LPS versus control); ##*P* < 0.01 and #*P* < 0.05 (control + LPS versus pY-STAT3^mut^); ns indicates not significant

To rule out side effects of the inhibitor, we genetically suppressed pY-STAT3 through the Y705-STAT3 mutation. As expected, the LPS-induced proliferation of pY-STAT3 mutant macrophages was successfully limited (Fig. 5a). We found decreased secretion of cell supernatant IL-6, TNF-α and TF in the cell supernatants of LPS-stimulated pY-STAT3 mutant macrophages compared with LPS-stimulated control macrophages (Fig. 5B). Similarly, the fluorescence intensity of TF in LPS-challenged pY-STAT3 mutant macrophages was reduced (Fig. 5c). In addition, RT-PCR analysis revealed a decline in the mRNA expression of TNF-α, IL-1β, IL-6, IL-10, MCP1, CXCL1, CXCL10, MMP9 and TF in LPS-stimulated pY-STAT3 mutant macrophages (Fig. 5d). We also found that the hyperactivation of the JAK2/STAT3, NF-κB, MAPK and AKT pathways in pY-STAT3 mutant macrophages challenged by LPS was effectively curbed (Fig. 5e). In short, these findings verified that the targeted suppression of pY-STAT3 is capable of modulating the macrophage inflammatory response and TF expression via the JAK2/STAT3, NF-κB, MAPK and AKT signaling pathways.

### TLR4 modulates pY-STAT3-associated inflammatory responses in vitro and in vivo

**Fig. 6:**
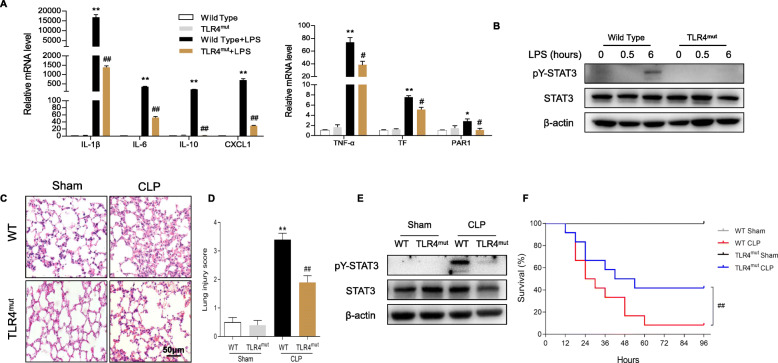
TLR4 regulates inflammation through STAT3 signaling in vitro and in vivo. PMs from TLR4^mut^ mice or wild type mice were challenged with LPS for 0.5 or 6 h. **a** The mRNA expression levels of the indicated genes were measured by RT-PCR (*n* = 4 per group; results are the mean ± SEM; one-way ANOVA test). **b** Immunoblotting for STAT3 signaling molecules in macrophages (*n* = 3 per group). **c**, **d** Lung tissue H&E staining (**c**, *n* = 4 per group; scale bar: 50 μm) and lung injury score (**d**, *n* = 10). **e** Immunoblot analysis of STAT3 expression in lung tissues (*n* = 3 per group). **f** Survival analysis at 96 h (*n* = 12 mice per group; Kaplan-Meier survival analysis). **P < 0.01 and *P < 0.05 (wild type versus wild type + LPS; WT CLP versus TLR4^mut^ CLP); ##*P* < 0.01 and #*P* < 0.05 (wild type + LPS versus TLR4^mut^ + LPS; WT CLP versus TLR4^mut^ CLP)

Based on our results that pY-STAT3 was related to the NF-κB pathway in macrophages, we hypothesized that there is crosstalk between TLR4 and STAT3 signaling. We next extracted peritoneal macrophages (PMs) from TLR4 mutant mice. Upon LPS stimulation, TLR4 mutant PMs expressed obviously lower mRNA levels of inflammatory mediators (TNF-α, IL-1β, IL-6, IL-10 and CXCL1), TF and PAR1 (Fig. 6a). The immunoblot revealed a weaker activation of STAT3 in LPS-challenged TLR4 mutant PMs (Fig. 6b). These proved a crosstalk between TLR4 and STAT3 pathway in LPS-induced macrophages. We also explored the effects of CLP in TLR4 mutant mice and found that TLR4 mutant CLP mice had milder lung injury than wild type CLP mice (Fig. 6c, d). The immunoblots suggested that TLR4 mutant CLP mice had lower levels of pY-STAT3 in lung tissue (Fig. 6e). In addition, the mutation of TLR4 prolonged animal survival in the CLP model (Fig. 6f).

## Discussion

Sepsis ia a serious problem worldwide, incurring the greatest hospital costs to hospital and giving rise to most deaths in intensive care units [33]. Clearly, the activation of pattern recognition receptors (PPRs) in response to microbial pathogens triggers the imbalance of the inflammation-coagulation network, leading to death during sepsis [34]. Consequently, elucidating the key signaling molecules of this network can provide a promising therapeutic strategy for sepsis. Our findings demonstrated that pY-STAT3 is an effective therapeutic target in regulating inflammation and coagulation activation, which contributes to the pathogenesis of sepsis.

STAT3 is a pleotropic transcription factor that mediates multiple biological activities, including inflammation, metabolism and development [35]. STAT3 has previously been shown to be activated by phosphorylation at tyrosine 705 and serine 727 following stimulation by TLR agonists, cytokines and growth factors [36]. Our studies found that STAT3 was hyperactive at tyrosine 705 in the lung tissues of CLP-induced septic mice. Previous studies have reported that STAT3 inhibitors have effects on cancers, such as pancreas and esophagus cancers [37]. Therefore, the total STAT3 inhibitor Na and the STAT3 phospho-Tyr705 selective inhibitor BP were chosen as treatments for CLP mice. The results showed that BP, but not Na, efficaciously protected mice against septic fatal death, marked inflammation and defective coagulation which indicated the potential role of pY-STAT3 in sepsis. Since STAT3-deficient mice are embryonic lethal, conditional tissue-specific gene knockout strategies were applied for STAT3 study in different diseases. Macrophage/neutrophil-specific STAT3 deletion mice were more susceptible to endotoxemia and sepsis associated with higher systemic inflammation, weaker bacterial clearance, more severe multiple organ dysfunction and increased mortality [30]. Our findings also verified that the total STAT3 inhibitor could not rescue the mice from septic death.

Coagulopathy is a critical host response to infection accompanied by an inflammatory response that can result in disseminated intravascular coagulation (DIC) with increased mortality during sepsis [38]. DIC, featured by the systemic overactivation of coagulation, is associated with intravascular thrombosis formation that subsequently leads to multiple organ dysfunction [8, 18]. The products and stimuli from microbes induce TF secretion, a potent initiated thrombin activator mainly produced by monocytes, epithelial cells and endothelial cells, to promote septic coagulopathy, followed by increasing the plasma levels of TAT, D-Dimer and PAI1 [39, 40]. Our results suggested that pY-STAT3 inhibition suppressed the production of TF, TAT and D-Dimer in septic mice. However, the increased PAI1 level was not affected by the treatment. We hypothesized that interference of pY-STAT3 had no effect on the regulation of the fibrinolytic system.

ALI is one of the most important organ injuries caused by sepsis and has complex mechanisms, including cytokine storm and pulmonary capillary leakage [41–43]. Previous studies have shown that Stattic and LLL12 were utilized to suppress STAT3 activity and ameliorate inflammatory responses in LPS-induced ALI and LPS-challenged macrophages [29]. Our results also supported the notion that the pY-STAT3 inhibitor BP had obvious effects on pulmonary protection. Vascular inflammation and hyperpermeability are common features of the pathogenesis of sepsis, and pulmonary endothelial cell dysfunction plays a key part in sepsis-induced ALI [44–46]. Numerous studies have shown that STAT3 signaling is involved in endothelial cell function [47–49]. However, the detailed mechanism of cell permeability is still unclear. In the present study, we revealed that BP administration stabilized HPMECs integrity by affecting the localization of VE-cadherin and α-E-catenin. Angiogenesis which indicates the imbalance of endothelial cells, was impaired by BP under LPS stimulation. We found that the LPS-induced HPMECs tube formation was restrained by pY-STAT3 inhibition. Coagulation activation-associated factors are also closely related to endothelial cell damage including TF, TM, EPCR and PAI1. Previous studies have found that the targeted therapies for these coagulation indicators had protective effects on sepsis [4, 6]. Nevertheless, our study discovered that the pY-STAT3 inhibitor BP could not improve the abnormal TF, TM, EPCR and PAI1 levels in HPMECs by LPS priming.

The abnormal changes in the cellular biological characteristics of the inflammatory response and cell growth exhibit homeostatic imbalances during sepsis [50]. We found that the proinflammatory factor production and cell proliferation were suppressed by pY-STAT3 inhibition in vivo and in vitro. The proliferation of macrophages stimulated by LPS was controlled by pharmacological or genetic pY-STAT3 inhibition. The immunofluorescence double staining with Ki67 and F4/80 for proliferating macrophages showed that the increased lung macrophage infiltration in CLP mice was effectively restrained by BP treatment.

The IL-6/JAK2/STAT3, NF-κB, MAPK and AKT pathways are central signaling pathways in pathophysiological processes and interact with each other in inflammatory diseases [22, 51, 52]. However, the crosstalk between these signaling pathways in sepsis is not fully understood. Our results proved that the inhibition of STAT3 activation can decrease hyperinflammatory factor production in cell and animal experiments through the JAK2/STAT3 (p-JAK2 and pY-STAT3), NF-κB (p-IKKα/β, p-IκBα and p-P65), MAPK (p-JNK, p-Jun, p-P38 and p-ERK1/2) and AKT (p-AKT) pathways in an interactive way. The TF gene expression by LPS induction in macrophages and endothelial cells requires the activation of various transcription factors, including NF-κB, MAPK, Egr-1 and protease activated receptors (PARs) [53–56]. In addition, to our knowledge, our study is the first to find that TF expression is regulated by STAT3 signaling with LPS stimulation, which may provide a new molecular mechanism for the diseases mediated by TF. To verify whether STAT3 has a connection with TLR4, we extracted PMs from TLR4 mutant mice and found that the TLR4-mutant macrophages showed reduced activation of STAT3 and production of inflammatory and coagulant factors following LPS challenge. Moreover, we also explored the effects of CLP on TLR4 mutant mice and found that TLR4 mutant CLP mice have milder lung injury, lower STAT3 activation and a higher survival rate than wild type CLP mice.

While our results showed that pY-STAT3 is a promising therapeutic target in sepsis, the role of STAT3 as an essential transcription factor makes the development of pSTAT3-targeted therapies challenging. Further in-depth analysis of the regulatory function of the STAT3 pathway in sepsis will be the focus of future research. We should also focus on the role of negative regulators of the STAT3 pathway in the treatment of sepsis.

## Conclusions

**Fig. 7:**
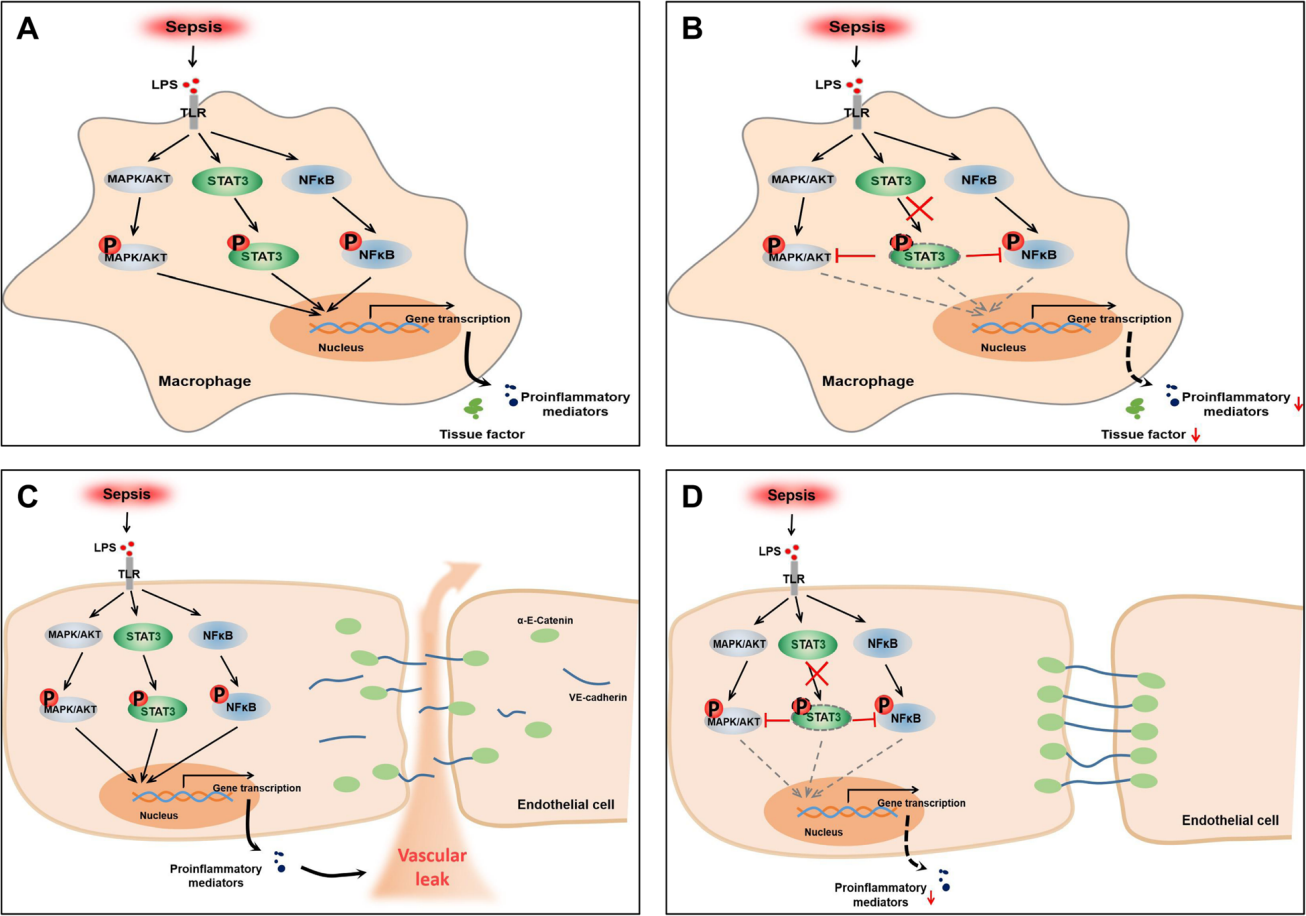
Schematic of the regulation of septic inflammation and coagulopathy in endothelial cells and macrophages by STAT3 signaling. **a**, **b** In macrophages, inhibition of pY-STAT3 reduces sepsis-induced proinflammatory mediators and TF expression through inactivation of JAK2/STAT3, NF-κB, MAPK and AKT signaling. **c**, **d** In endothelial cells, inhibition of pY-STAT3 protects cells from inflammatory injury and hyperpermeability via JAK2/STAT3, NF-κB, MAPK and AKT pathways and enhances cellular VE-cadherin/α-E-catenin localization

In summary, as shown in Fig. 7, our study revealed the crucial role of activated STAT3 in inflammation and coagulation in sepsis. The inhibition of STAT3 activation can markedly limit the hyperactivation of the inflammatory response, suppress the coagulation activation, and protect the endothelial barrier function through the MAPK, AKT, STAT3 and NF-κB pathways which promote each other interactively. Therefore, targeting pY-STAT3 presents a potential therapeutic strategy for combating sepsis.

## Supplementary information

**Additional file 1.**

## Data Availability

All data generated or analysed during this study are included in this published article.

## Abbreviations

pY-STAT3: phospho-Tyr705 of STAT3
CLP: Cecal ligation and puncture
BP: BP-1-102
Na: Napabucasin
HPMECs: Human pulmonary microvascular endothelial cells
LPS: Lipopolysaccharide
TF: Tissue factor
TAT: Thrombin-antithrombin complex
ALI: Acute lung injury
TLR4: Toll-like receptor 4
PMs: Peritoneal macrophages
CCK-8: Cell Counting Kit-8
PAI1: Plasminogen activator inhibitor 1
EBD: Evans blue dye
TM: Thrombomodulin
EPCR: Endothelial protein C receptor
PPRs: Pattern recognition receptors
DIC: Disseminated intravascular coagulation
PARs: Protease activated receptors
