# Supplementary material for: Phospho-Tyr705 of STAT3 is a therapeutic target for sepsis through regulating inflammation and coagulation
Source: Cell Commun Signal. 2020 Jul 8;18:104. doi: 10.1186/s12964-020-00603-z (PMC7341624; doi:10.1186/s12964-020-00603-z)
Supplement: Supplementary file 2 — Additional file 1. [file 12964_2020_603_MOESM2_ESM.doc]

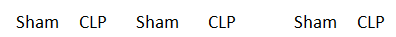
**Fig. 1**


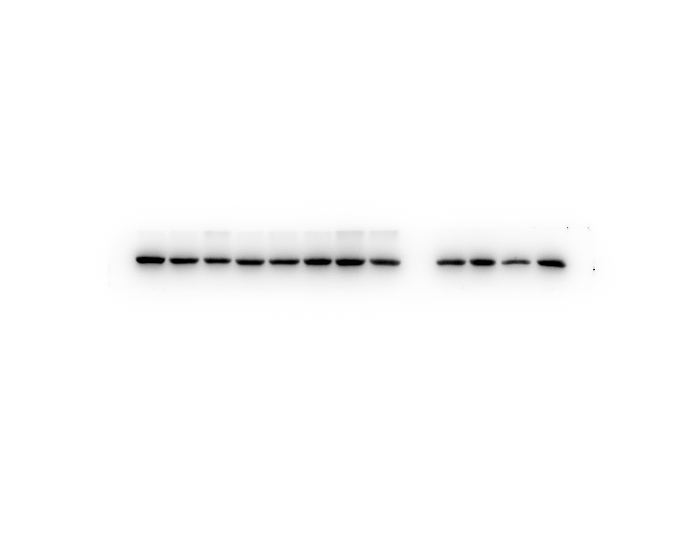

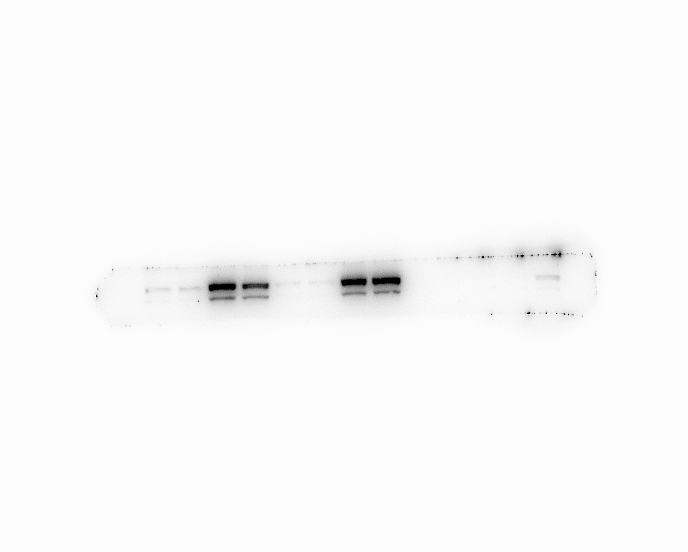

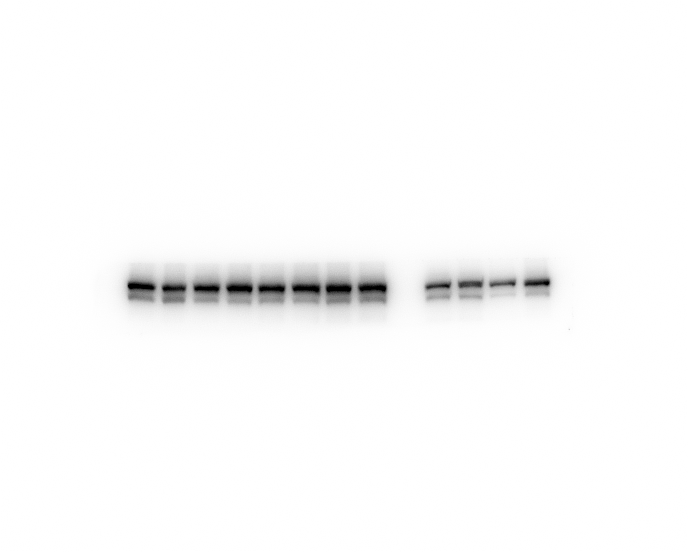


p-Y705 STAT3

STAT3

β-actin


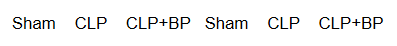
**Fig. 2**


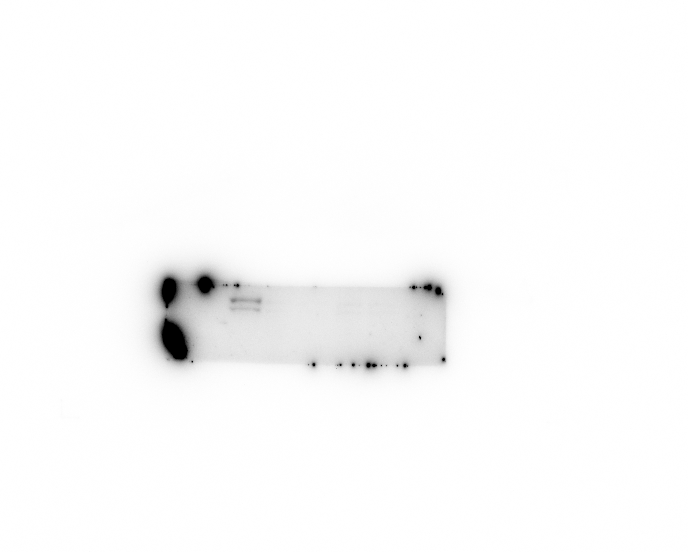


pY-STAT3


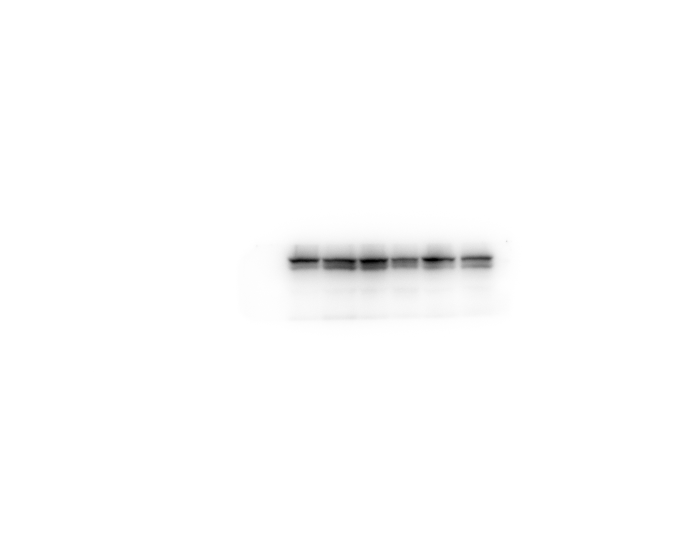


STAT3


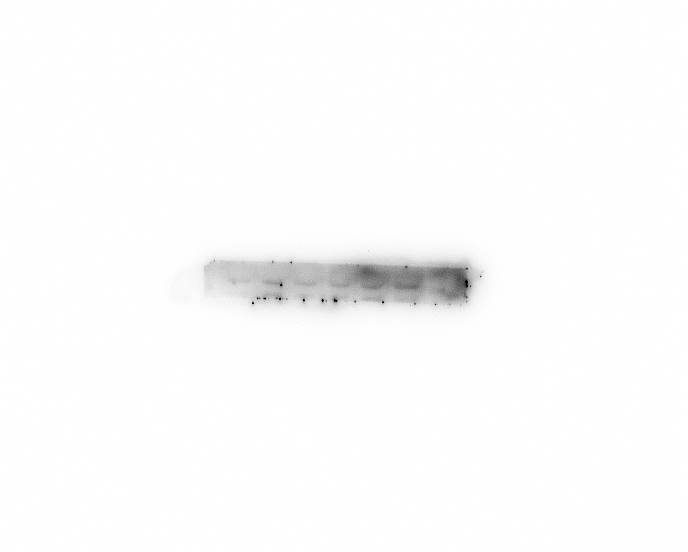


p-ERK1/2


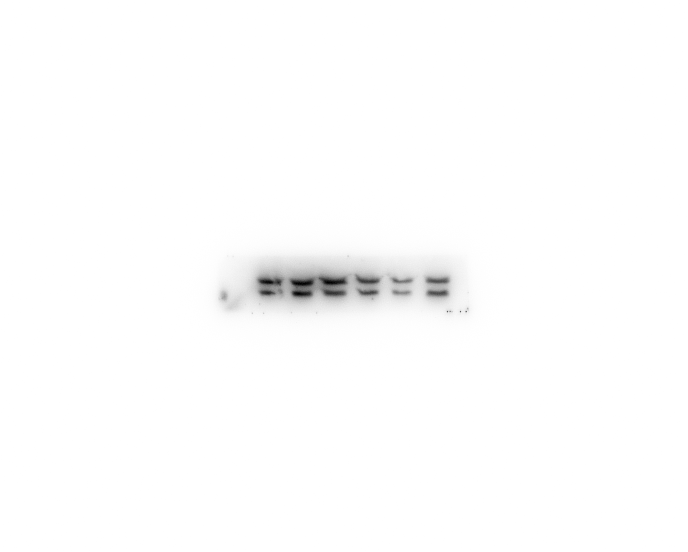


ERK1/2


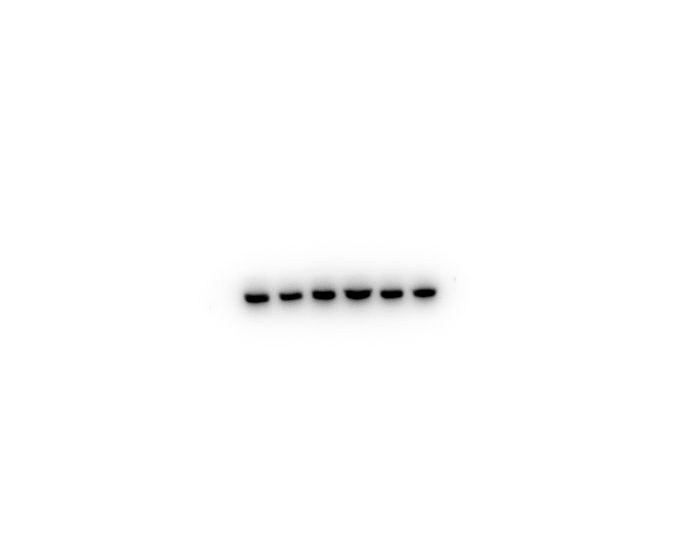


β-actin


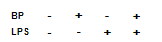
**Fig. 3**


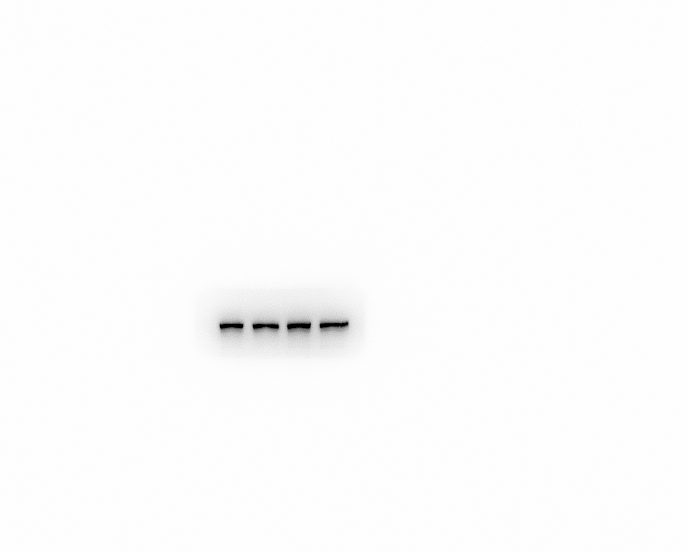


VE-cadherin


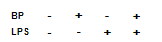


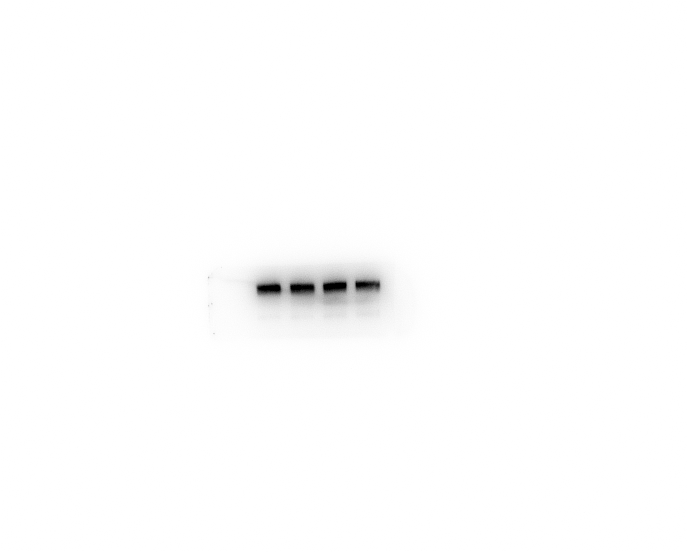


α-E-catenin


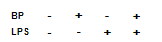


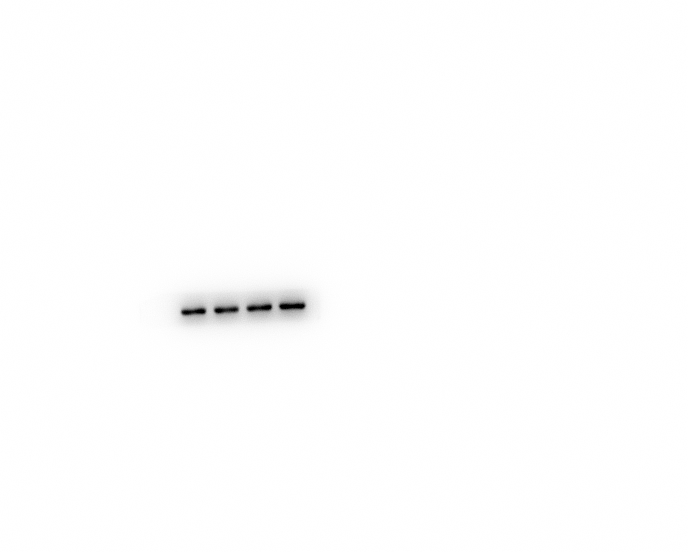


β-actin


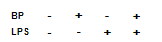


p-JNK


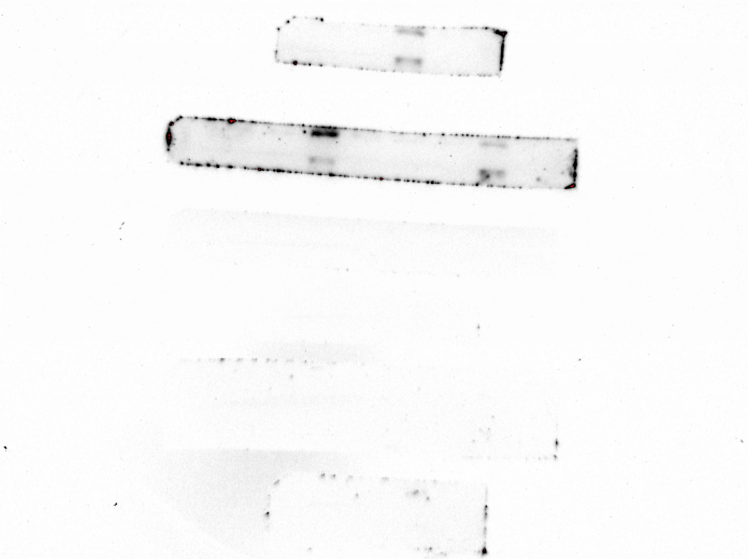


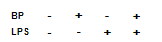


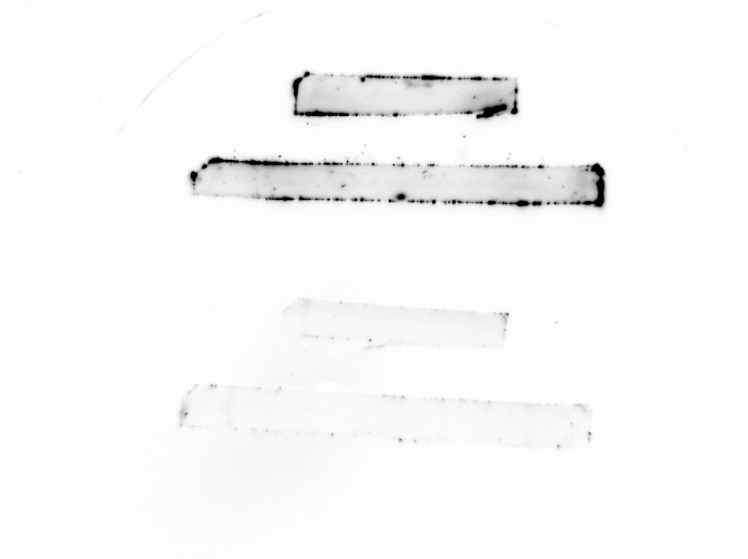


p-AKT


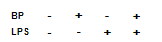


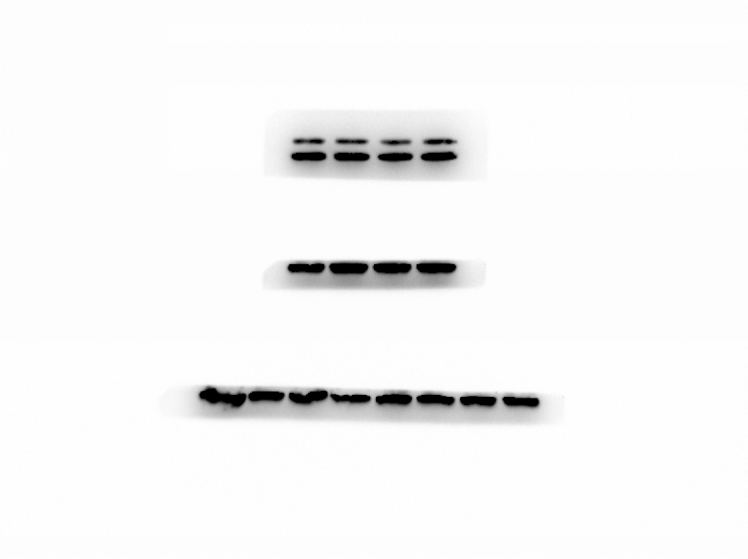
AKT


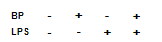


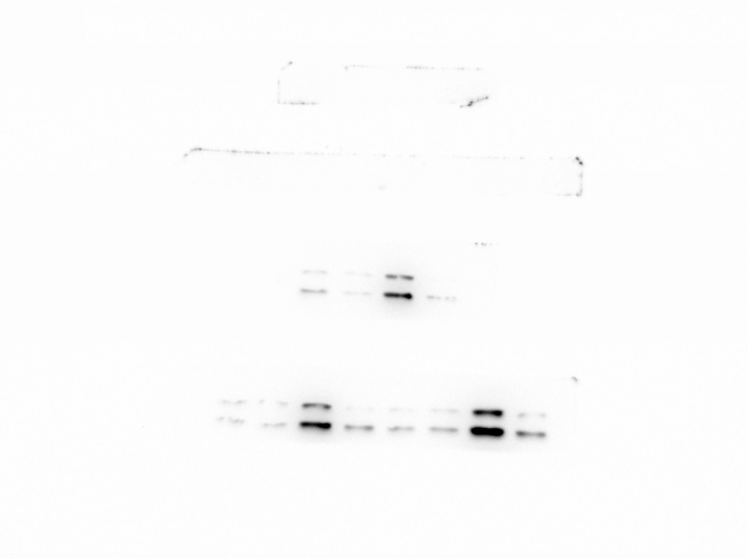
p-ERK1/2


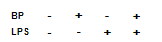


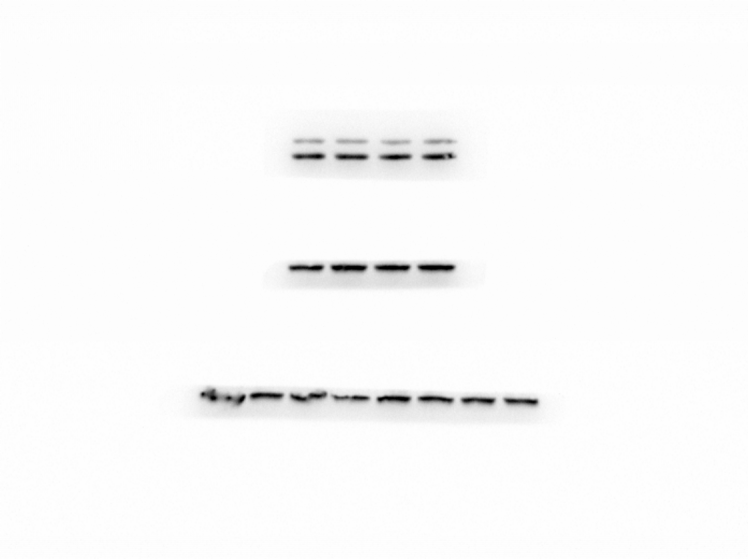
ERK1/2


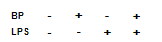

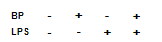


p-IκBα


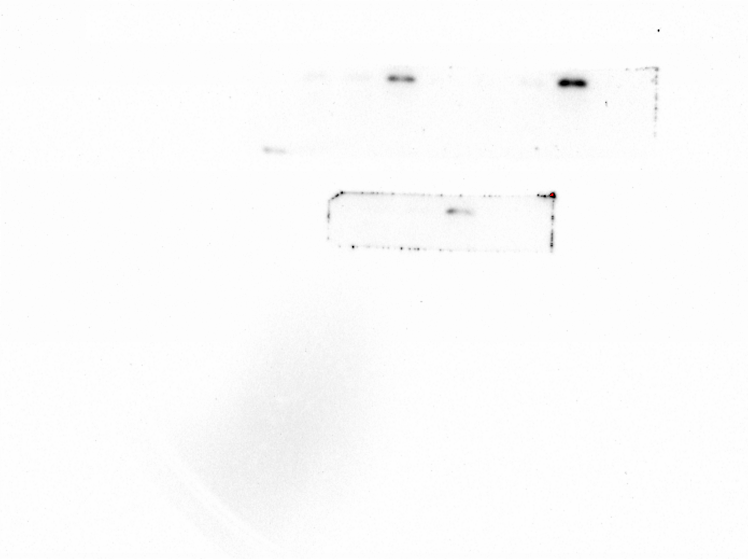


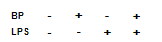

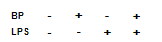


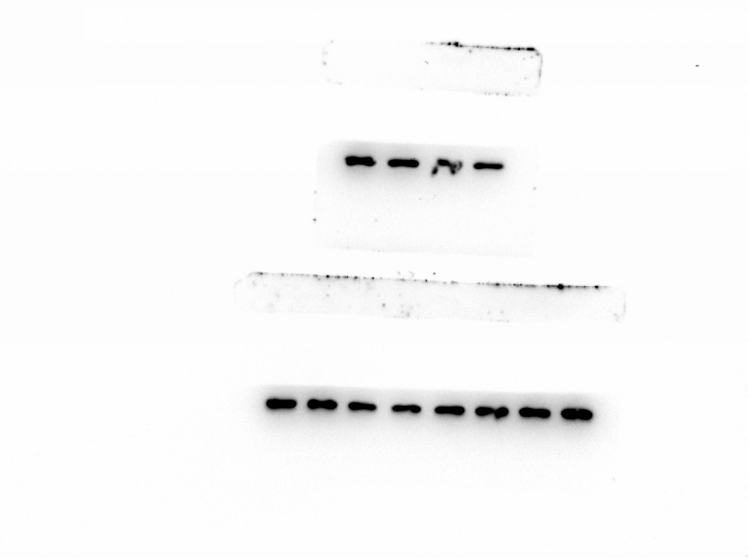
IκBα


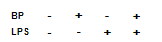


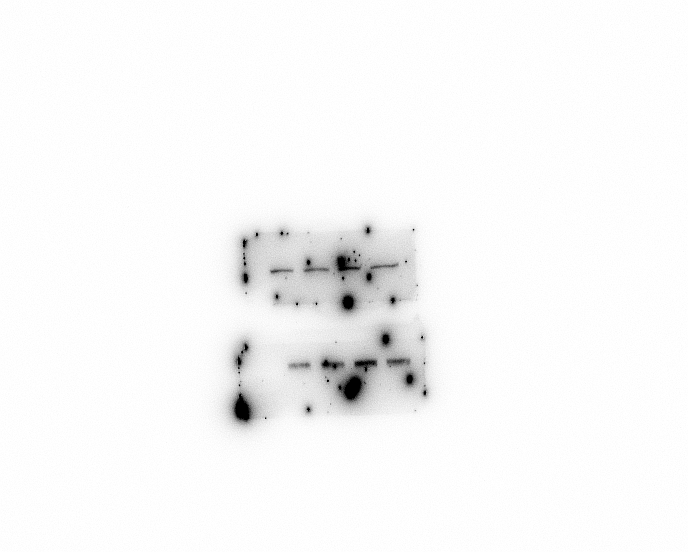


p-JAK2


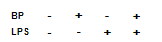


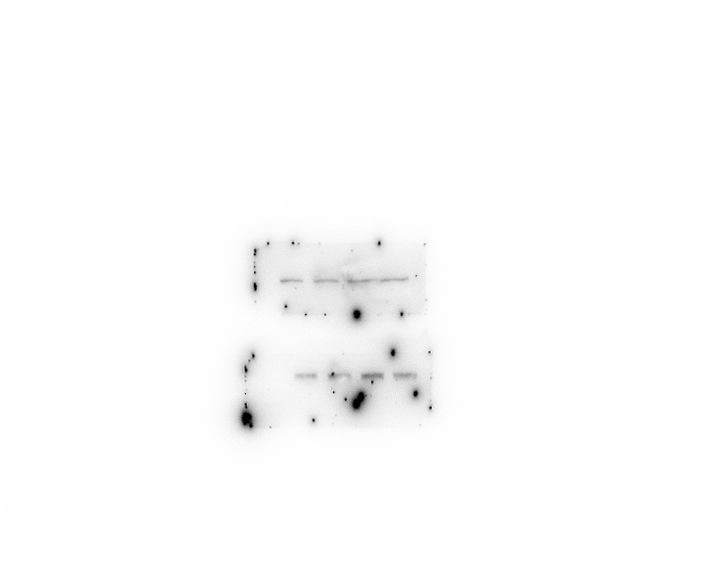


JAK2


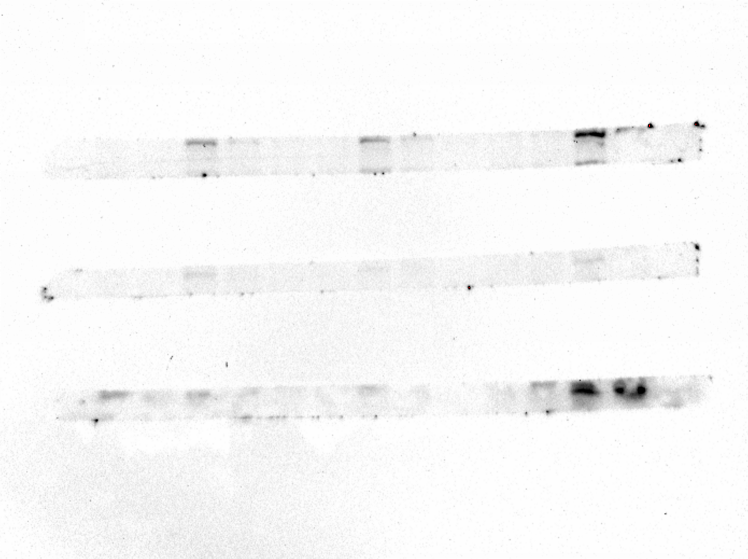


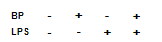

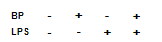

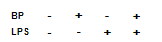


p-STAT3


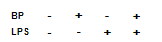

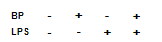

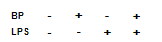


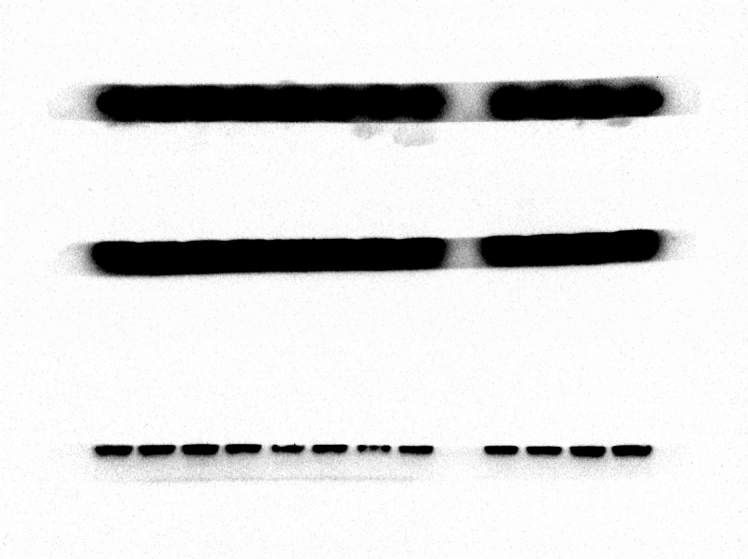


STAT3


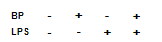

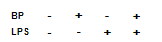

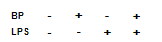


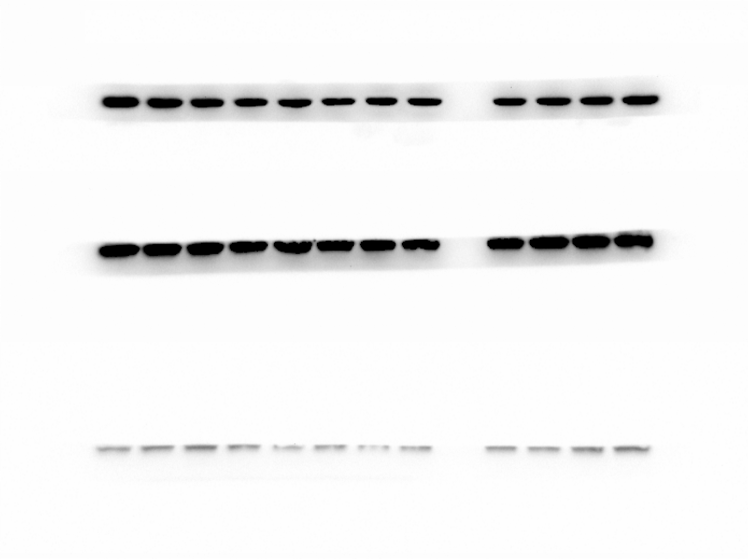


β-actin

**Fig. 4**


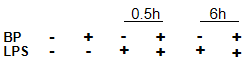
p-IKKα/β


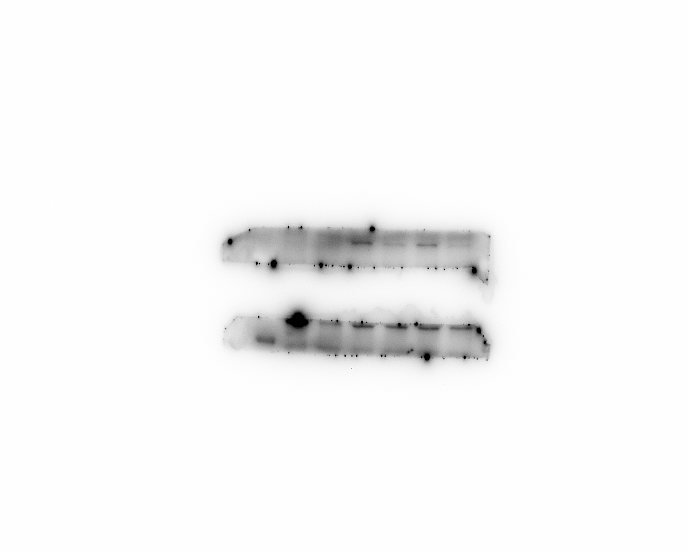


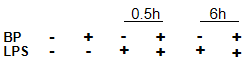


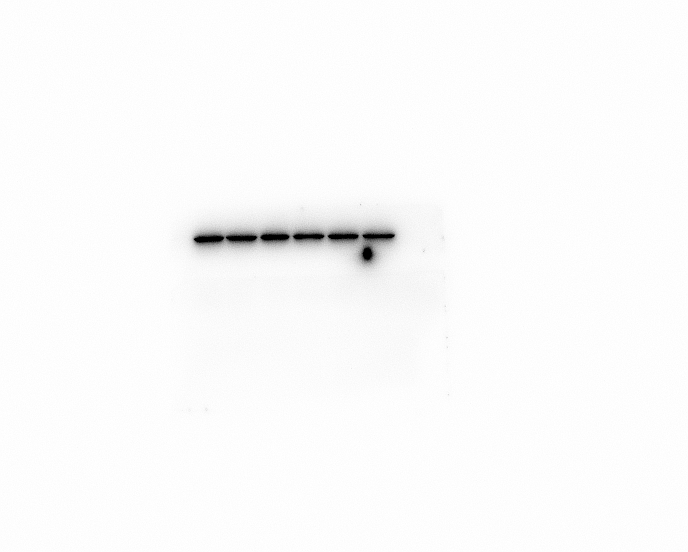


IKKβ


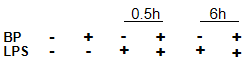


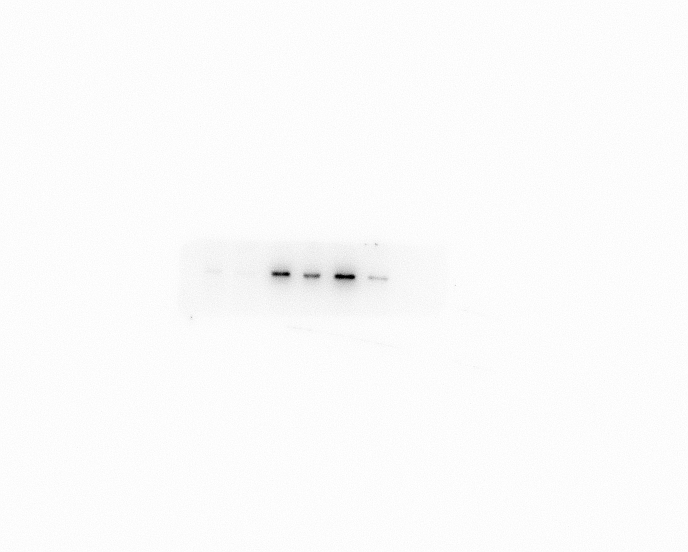


p-IκBα


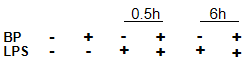


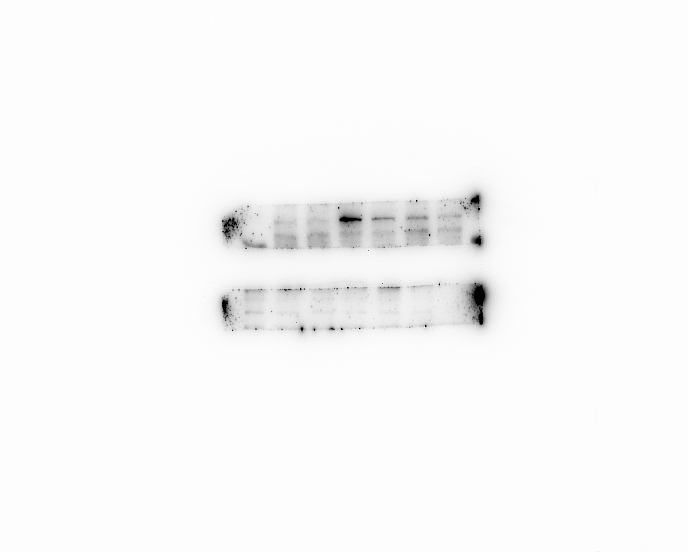


p-P65


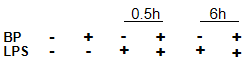


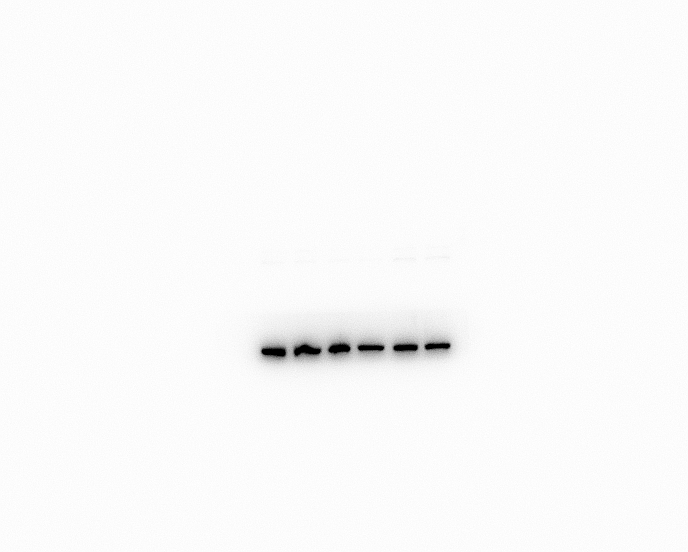


P65


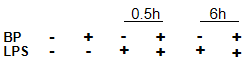


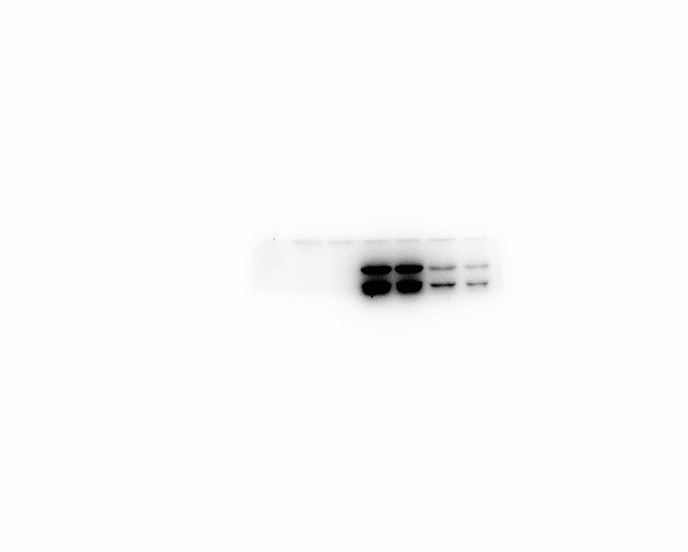
p-JNK


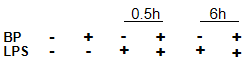


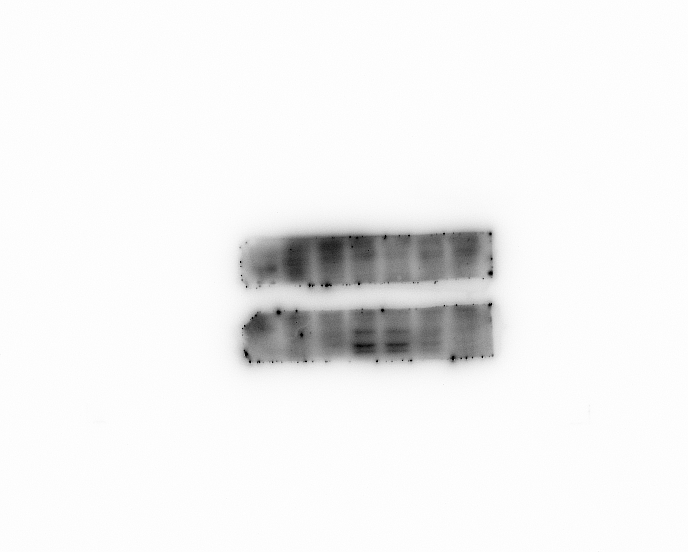


p-P38


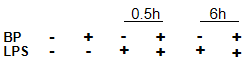
P38


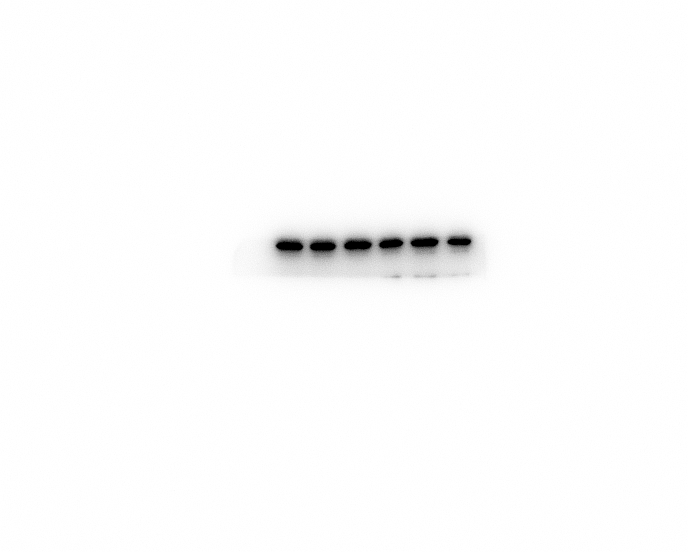


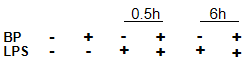


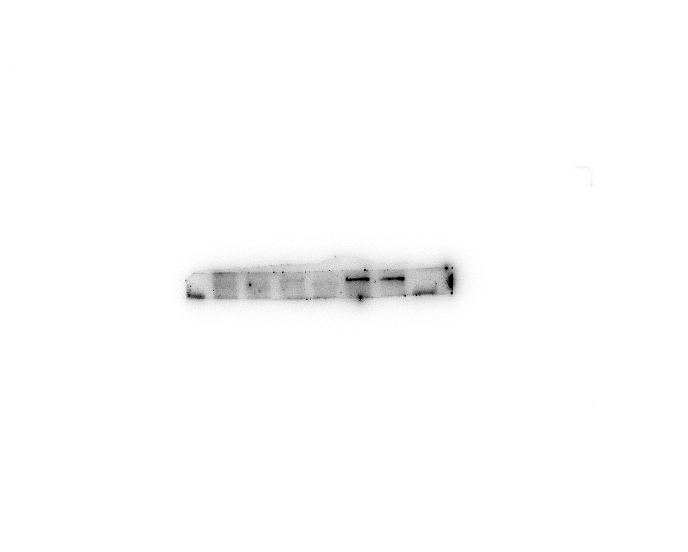
p-AKT


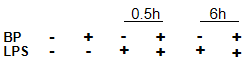


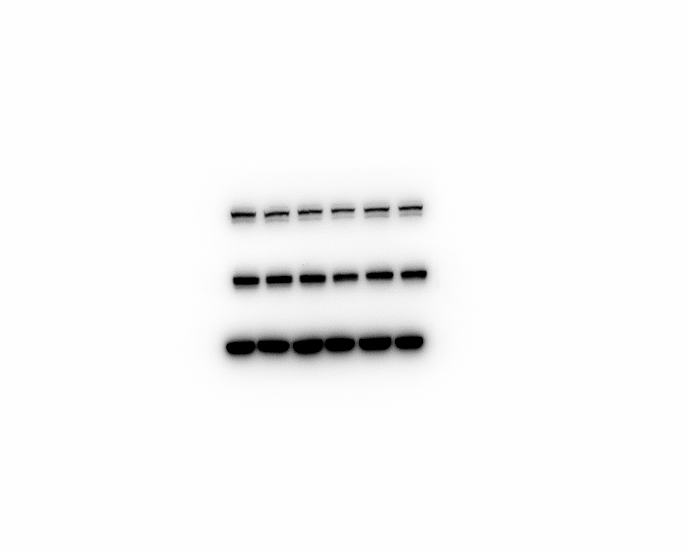


AKT


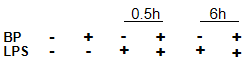


p-ERK1/2


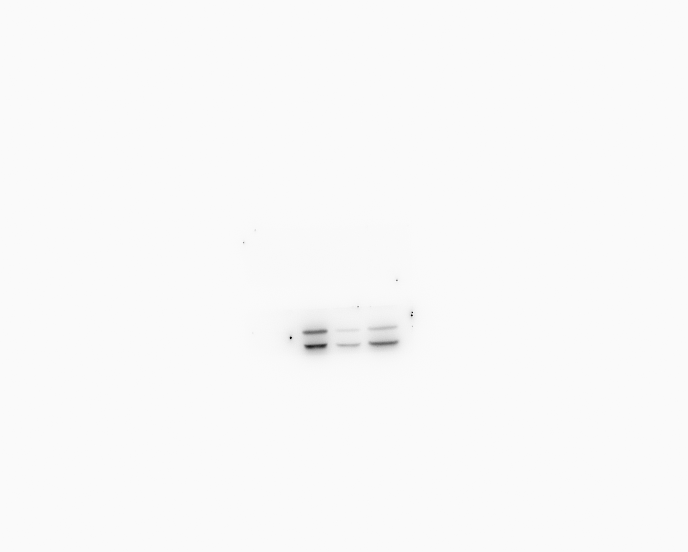


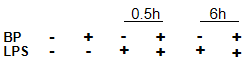


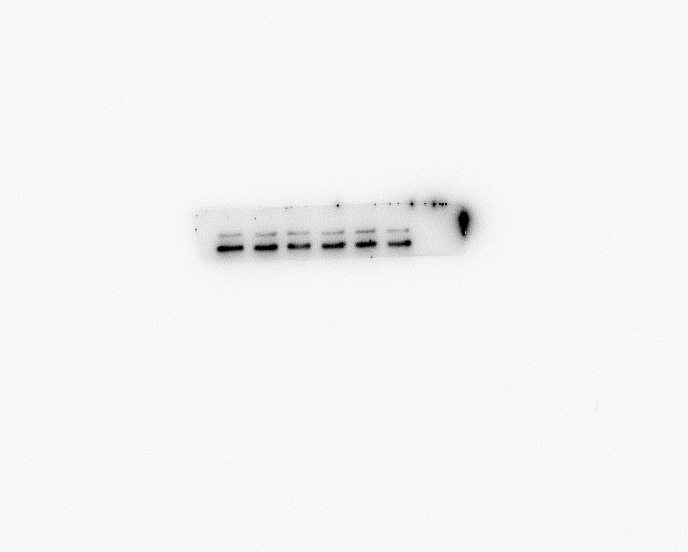


ERK


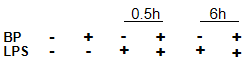


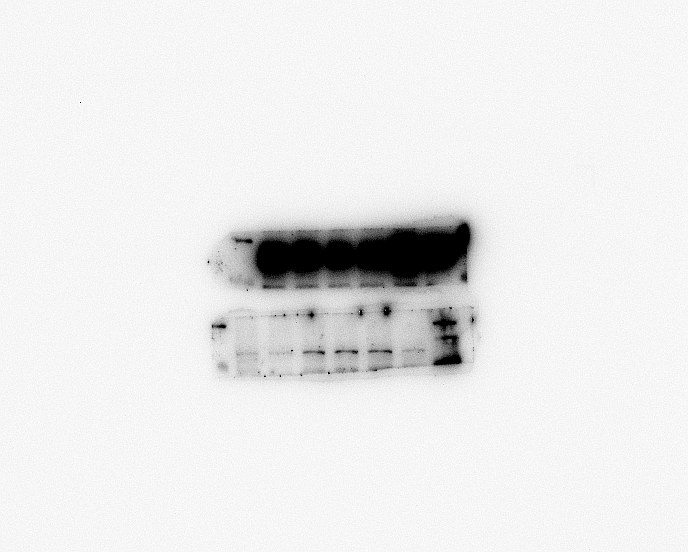


p-JAK2


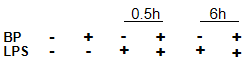


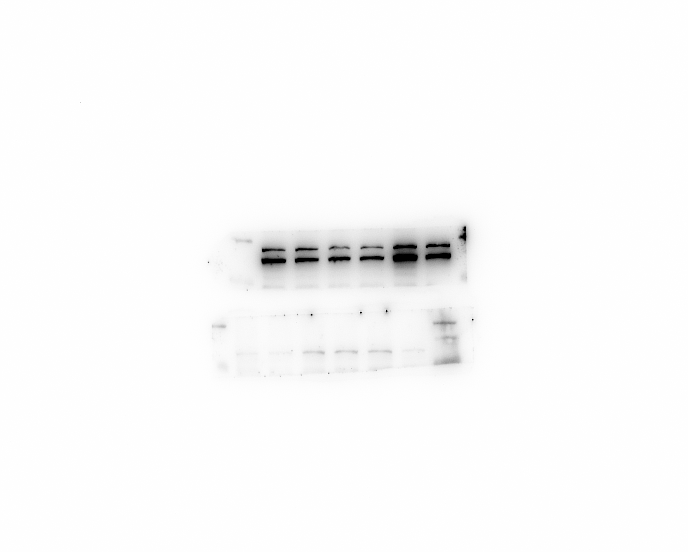


JAK2


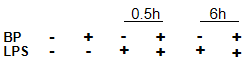


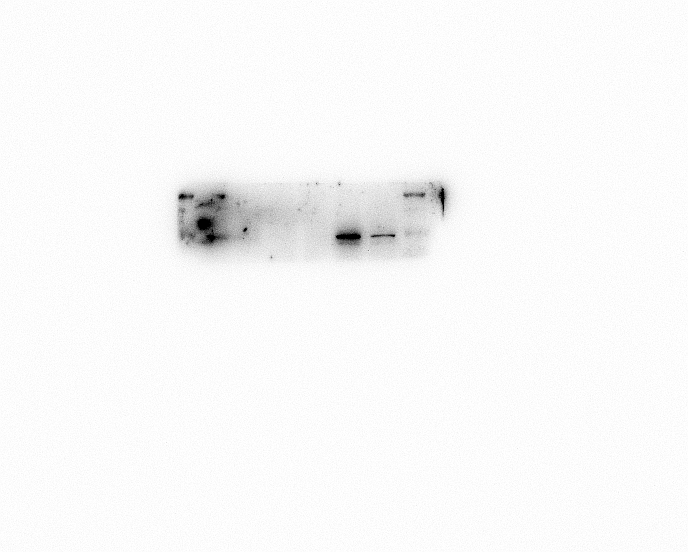


p-STAT3


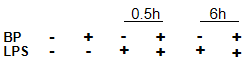


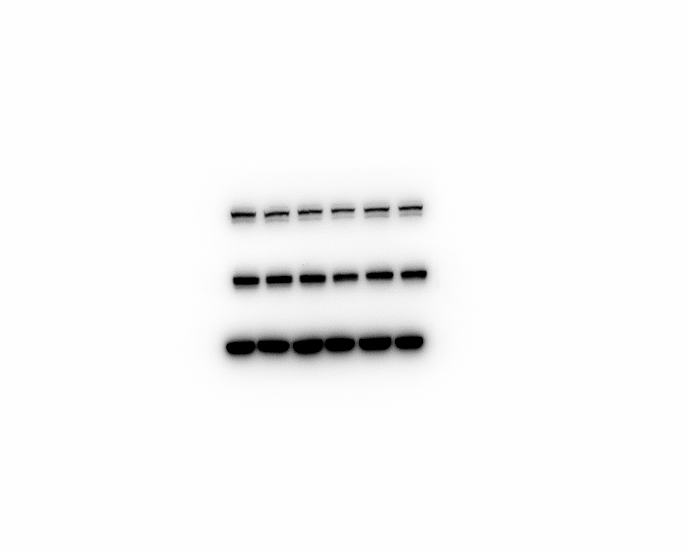
STAT3


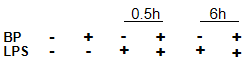


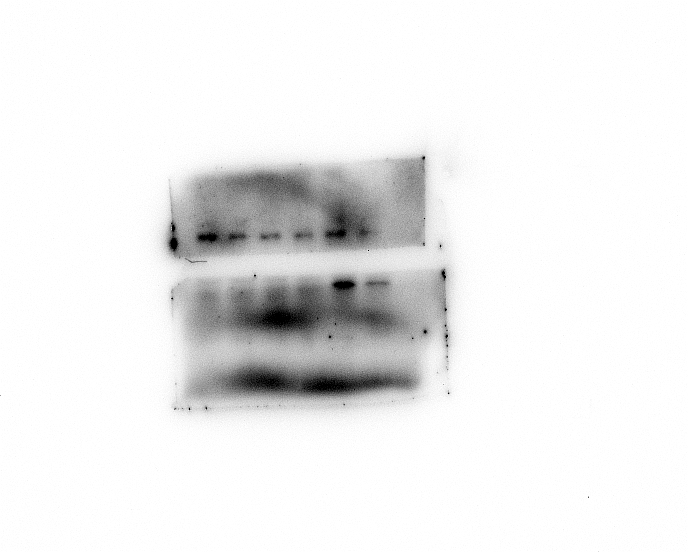


TNF-α


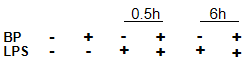


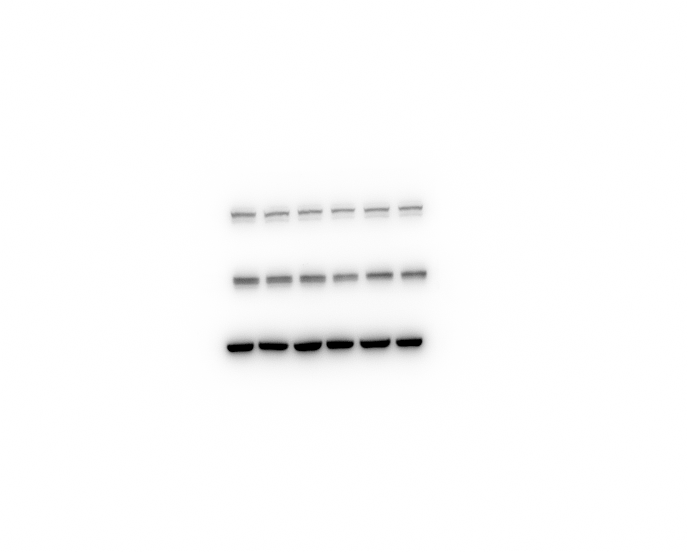
β-actin


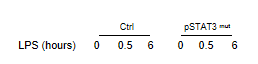
**Fig. 5**


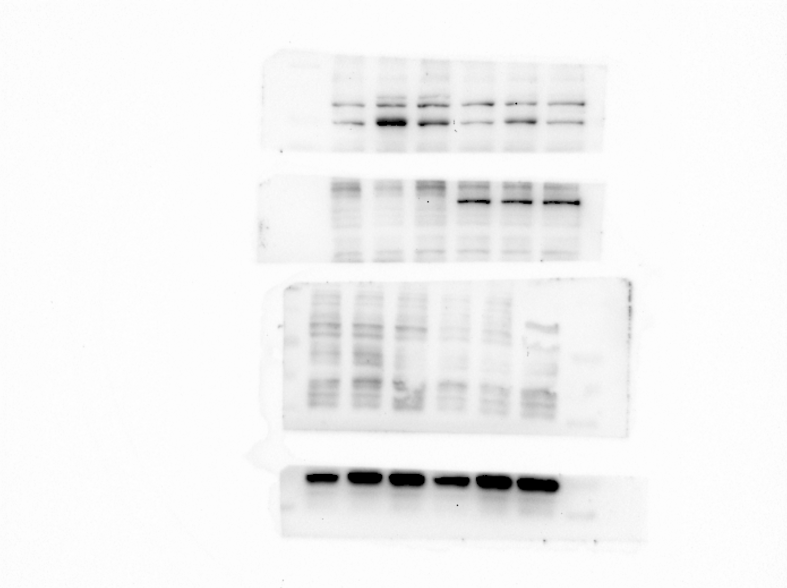
p-IKKα/β


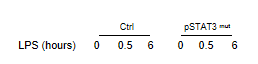


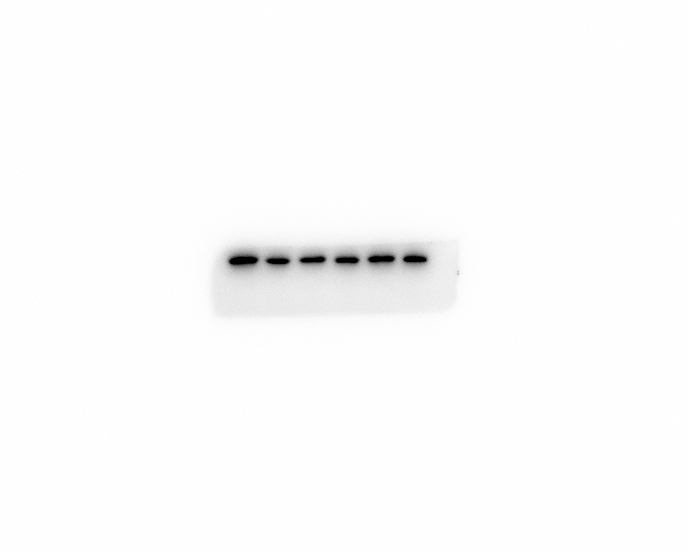


IKKβ


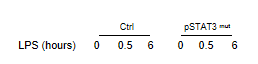


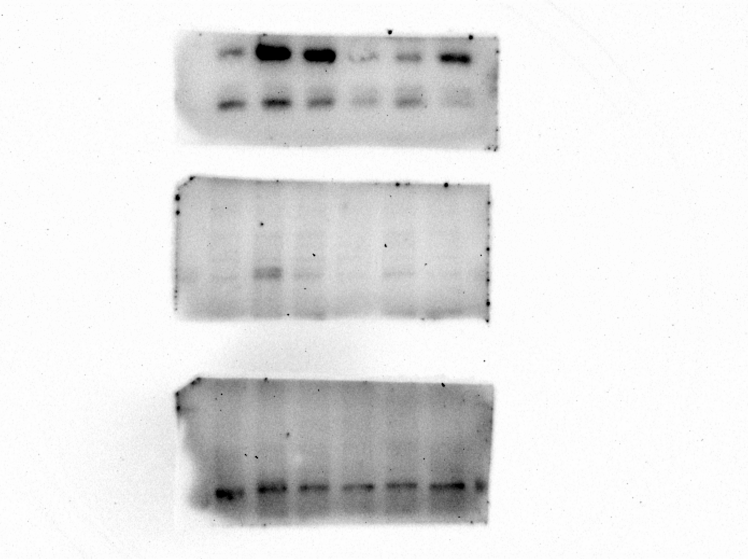
p-IκBα


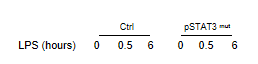


I
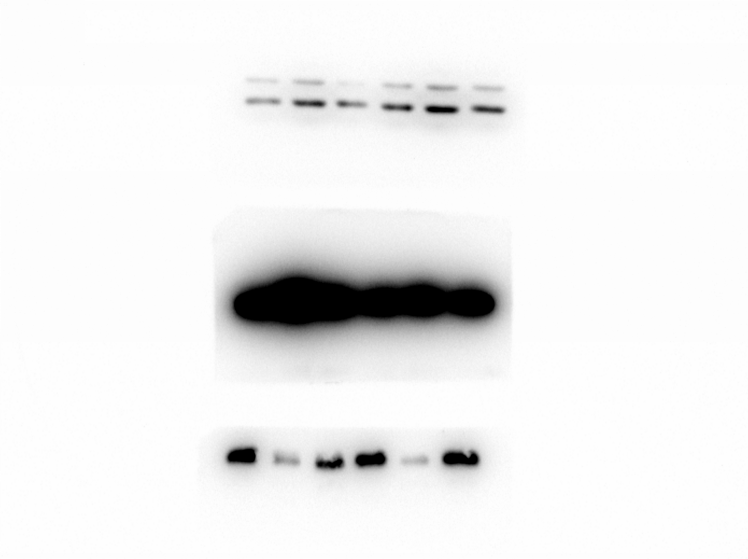
κBα


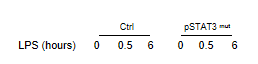


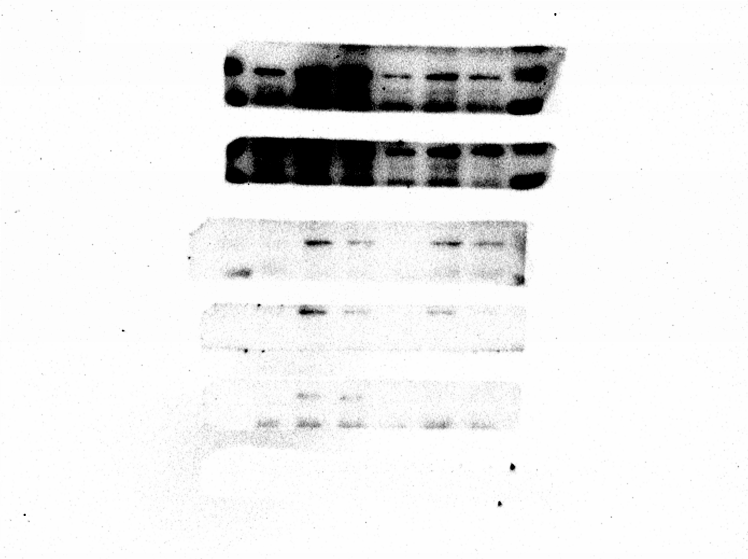
p-P65


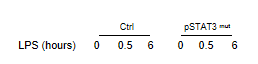


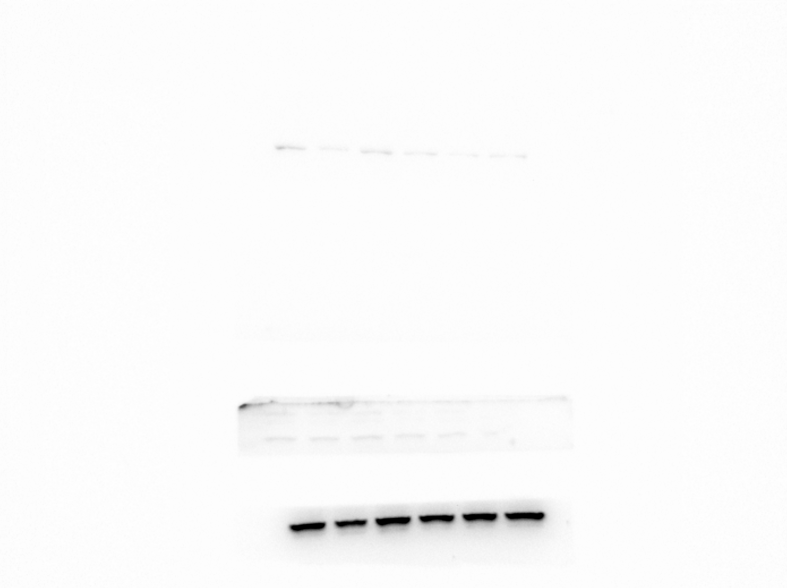


P65


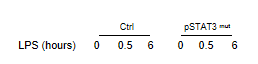


p-JNK


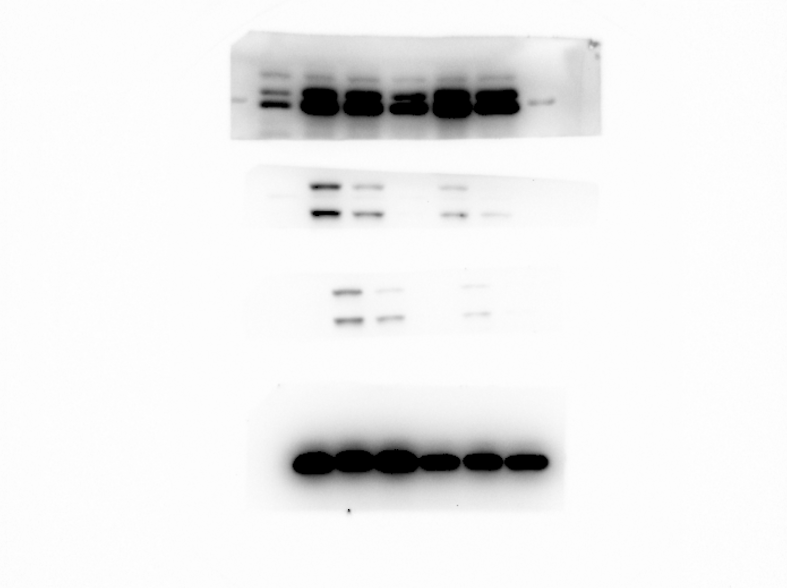


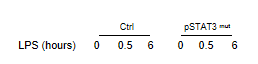


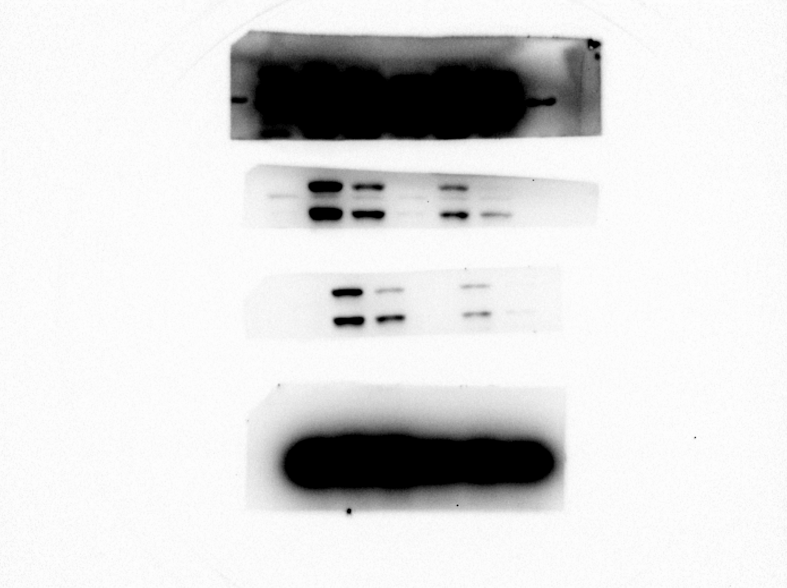
c-Jun

p-P38

P38

p-AKT

AKT

p-ERK1/2

ERK1/2

p-JAK2

JAK2

p-STAT3

STAT3

TNF-α

β-actin

**Fig. 6**

p-STAT3

STAT3

β-actin
